# 2-Methoxyestradiol protects against pressure overload-induced left ventricular hypertrophy

**DOI:** 10.1038/s41598-018-20613-9

**Published:** 2018-02-09

**Authors:** Zaid H. Maayah, Jody Levasseur, Ramanaguru Siva Piragasam, Ghada Abdelhamid, Jason R. B. Dyck, Richard P. Fahlman, Arno G. Siraki, Ayman O. S. El-Kadi

**Affiliations:** 1 Faculty of Pharmacy & Pharmaceutical Sciences, Katz Group-Rexall Centre for Pharmacy and Health Research, University of Alberta, Edmonton, T6G 2E1 Canada; 2 Cardiovascular Research Centre, Department of Pediatrics, Mazankowski Alberta Heart Institute, Faculty of Medicine and Dentistry, University of Alberta, Edmonton, Alberta Canada; 3 Department of Biochemistry, Faculty of Medicine & Dentistry, University of Alberta, Edmonton, Canada; 4 Department of Oncology, Faculty of Medicine & Dentistry, University of Alberta, Edmonton, Canada

## Abstract

Numerous experimental studies have supported the evidence that 2-methoxyestradiol (2 ME) is a biologically active metabolite that mediates multiple effects on the cardiovascular system, largely independent of the estrogen receptor. 2 ME is a major cytochrome P450 1B1 (CYP1B1) metabolite and has been reported to have vasoprotective and anti-inflammatory actions. However, whether 2 ME would prevent cardiac hypertrophy induced by abdominal aortic constriction (AAC) has not been investigated yet. Therefore, the overall objectives of the present study were to elucidate the potential antihypertrophic effect of 2 ME and explore the mechanism(s) involved. Our results showed that 2 ME significantly inhibited AAC-induced left ventricular hypertrophy using echocardiography. The antihypertrophic effect of 2 ME was associated with a significant inhibition of CYP1B1 and mid-chain hydroxyeicosatetraenoic acids. Based on proteomics data, the protective effect of 2 ME is linked to the induction of antioxidant and anti-inflammatory proteins in addition to the modulation of proteins involved in myocardial energy metabolism. *In vitro*, 2 ME has shown a direct antihypertrophic effect through mitogen-activated protein kinases- and nuclear factor-κB-dependent mechanisms. The present work shows a strong evidence that 2 ME protects against left ventricular hypertrophy. Our data suggest the potential of repurposing 2 ME as a selective CYP1B1 inhibitor for the treatment of heart failure.

## Introduction

Mechanisms regulating cardiac hypertrophy have been the focus of intense investigation in recent years. Among these mechanisms, cytochrome P450 (CYP) enzymes have been shown to play an important role in the regression or the progression of cardiac hypertrophy through the metabolism of arachidonic acid (AA) into cardioprotective epoxyeicosatrienoic acids (EETs) and cardiotoxic hydroxyeicosatetraenoic acids (HETEs)^1^. Of particular interest in the current study, CYP1B1 has been reported to contribute to the development of cardiovascular diseases for example ischemic heart diseases, hypertension, atherosclerosis, cardiac hypertrophy, and heart failure^2,3^. Mechanistically, CYP1B1 contributes to cardiovascular disease through oxidation of AA into cardiotoxic mid-chain HETEs metabolite and the formation of superoxides^1,4,5^.

Several studies have linked the increase in the formation of mid-chain HETEs metabolite to the development of cardiac hypertrophy and fibrosis. For example, 12-HETE was shown to induce cellular hypertrophy in cardiac fibroblasts^6^ whereas, 15-HETE has been reported to increase the sensitivity of the β-adrenergic response to ISO in cardiac cells^7^. The involvement of mid-chain HETEs in the development of heart failure was further investigated in transgenic mice that overexpressed 12/15-lipoxygenase (12/15-LOX) in cardiac tissues. The formation of 12- and 15-HETE metabolites over a relatively long period of time was associated with systolic dysfunction and cardiac fibrosis^8^. Furthermore, inhibiting the formation of 12- and 15-HETE significantly improved systolic dysfunction and prevented left ventricular dilatation in the presence of chronic pressure overload^8^.

In contrast to the negative effects of the cardiotoxic metabolites generated by CYP1B1, CYP1B1 also has a crucial role in the generation of cardioprotective metabolites, 2-methoxyestradiol (2 ME)^9^. This is supported by a recent finding demonstrated that Ang-II caused oxidative stress, cardiovascular changes, endothelial dysfunction and enhanced vascular reactivity in Cyp1b1(_−/−_) but not in Cyp1b1(_+/+_) female mice^9^. Furthermore, the cardiovascular remodeling induced by Ang-II coincided with a dramatic diminution in the formation of 2 ME in Cyp1b1(_−/−_) mice establishing a 2 ME-dependent mechanism. Moreover, when these mice were treated with 2 ME, the typical increase in blood pressure in Cyp1b1(−/−) mice did not occur, suggesting that not only 2 ME restored these knockout mice to normal levels, but also proposed that 2 ME may be a physiologically substantial metabolite at naturally occurring titers.

Interestingly, 2 ME was shown to exert a negative feedback effect on CYP1B1 catalytic activity^10^. Furthermore, 2 ME attenuates 2,3,7,8-tetrachloro dibenzo-p-dioxin-mediated oxidative stress by inhibiting CYP1B1 and its associated reactive metabolites^11^. Unlike TMS, a well-known CYP1B1 inhibitor, 2 ME protects against Ang II-induced hypertension and oxidative stress in female mice^9^. Furthermore, beneficial effects of 2 ME seem to be equal in males and females alike as it has few or no feminizing effects^12^.

The above observations not only propose using 2 ME as a promising candidate to treat left ventricular hypertrophy but it also implicates repurposing it as the best therapeutic approach to target CYP1B1. Accordingly, the current study was designed to: (1) explore the ability of 2 ME to protect against left ventricular hypertrophy *in vivo* in rats using AAC and *in vitro* in the human and rat cardiac cells and (2) examine the molecular mechanism(s) involved. Our findings may have substantial importance in understanding the beneficial effect of 2 ME against pressure-overload-induced cardiac hypertrophy and, therefore, introducing a new paradigm into the current pharmacopoeia using estrogenic metabolites as promising candidates to treat cardiovascular diseases.

## Materials and Methods

### Materials

2 ME, as well as the deuterated metabolites (internal standards), were purchased from Cayman Chemical (Ann Arbor, MI). Dulbecco’s Modified Eagle’s Medium/F-12 (DMEM/F-12), goat IgG peroxidase secondary antibody and 3-[4,5-dimethylthiazol-2-yl]-2,5-diphenyltetrazoliumbromide (MTT) were purchased from Sigma Chemical Co. (St. Louis, MO). TRIzol reagent was purchased from Invitrogen Co. (Grand Island, NY). High Capacity cDNA Reverse Transcription kit and SYBR^®^ Green PCR Master Mix were purchased from Applied Biosystems (Foster city, CA). Nitrocellulose was purchased from Bio-Rad Laboratories (Hercules, CA). CYP1B1 rabbit polyclonal (sc 32882), 5-LOX mouse monoclonal (sc-136195), 12-LOX rabbit polyclonal (sc-32939), 15-LOX mouse monoclonal (sc-133085), cyclooxygenase-2 (COX-2) mouse monoclonal (sc-376861) and glyceraldehyde-3-phosphate dehydrogenase (GAPDH) (sc 47724) mouse monoclonal primary antibodies in addition to anti-rabbit IgG peroxidase secondary antibody were purchased from Santa Cruz Biotechnology, Inc. (Santa Cruz, CA). The anti-mouse IgG peroxidase secondary antibody was purchased from R&D Systems (Minneapolis, MN, USA). PhosphoTracer ERK1/2, pT202/Y204, (ab176640), p38 MAPK, pT180/Y182, (ab176649) and JNK1/2/3, pT183/Y185, (ab176645) ELISA Kits were purchased from Abcam (Toronto, CA). NF-κB Family EZ-TFA Transcription Factor Assay Chemiluminescent Kit was purchased from Millipore (Millipore, Schwalbach/Ts., Germany, #70–660). ECL^TM^ Chemiluminescence western blot detection kits were obtained from GE Healthcare Life Sciences (Piscataway, NJ). All other chemicals were purchased from Fisher Scientific Co. (Toronto, ON).

### Animals

The study follows the Guide for the Care and Use of Laboratory Animals published by the US National Institutes of Health (Publication No. 85-23, eighth edition; revised 2011). The protocol of this study was approved by the University of Alberta Health Sciences Animal Policy and Welfare Committee. Male Sprague-Dawley rats, weighing 180–200 g, were purchased from Charles River Canada (St. Constant, QC, Canada). All animals were housed in cages under controlled environmental condition, a 12-hour light/dark cycle, and had free acess to food and water available ad libitum.

### Experimental design and treatment protocol

Male Sprague-Dawley rats of 12 weeks old, weighing 180–200 g were randomly assigned into four groups and were subjected to sham (n = 12) or AAC surgery (n = 12) to induce cardiac hypertrophy. The first group (n = 6) consisted of sham control rats that received polyethylene glycol (PEG 400) in mini osmotic pumps. The second group (n = 6) consisted of AAC rats that received polyethylene glycol in mini osmotic pumps. The third group (n = 6) consisted of sham 2 ME-treated rats that received 2 ME (5 mg/kg/day) in mini-osmotic pumps. The fourth group (n = 6) consisted of AAC rats that were treated with 2 ME as described in the aforementioned group.

All rats were anesthetized by isoflurane anesthesia (3% induction and 1–1.5% maintenance), disinfected with chlorohexidine soap and swab with betadine solution on the abdomen. Then a small incision was made through the skin beginning at the xyphoid sternum approximately 3–4 cm. The abdominal aorta was surgically dissected from the inferior vena cava at a site slightly above the renal arteries. A double-blunt needle was then placed along the side of the isolated aorta segment. The abdominal aorta was ligated with a syringe needle sized 21 G together by the silk suture sized 0. The needle was then removed, thus producing severe aortic constriction above the renal arteries. Visera was replaced carefully, abdominal wall was sutured and abdominal skin was closed with wound clips. The Sham procedure was performed as above with no ligation. One week after surgery, the osmotic mini-pumps were implanted subcutaneously under isoflurane anesthesia (3% induction and 1–1.5% maintenance).

Five weeks post-surgery, rats were echoed then euthanized under isoflurane anesthesia (3% induction and 1–1.5% maintenance) and hearts were quickly excised, washed with saline, blotted with filter paper and then the left ventricle was fragmented and homogenized to evaluate the mRNA, protein and metabolites level using a Branson homogenizer (VWR Scientific, Danbury, Conn., USA) whereas the other segment was fixed in 10% formalin for histopathology evaluation.

### *In vivo* evaluation of heart function by echocardiography and histopathology

Randomly selected animals from each group were anesthetized with isoflurane and transthoracic M-mode echocardiography (Vevo 770, Visualsonics, Toronto) was performed using a small animal imaging ultrasound system to measure cardiac function and wall thickness. Images were retained and analyzed using VisualSonics software version: 3.0.0. Left ventricular dimensions (LVD: left ventricular diameter; LVPW: left ventricular posterior wall thickness; and IVS: inter ventricular septum), left ventricular mass (LV mass), diastolic function and tissue droppler parameters in addition to ejection fraction (%EF) and fractional shortening (%FS) were determined using M-mode measurements taken from parasternal long and short axis views at the mid-papillary level. Left ventricular dimensions were recorded in systole and diastole. Pressure gradient (mmHg) was determined from the mitral flow using the velocity time intergral measurement. Measurements were averaged from 3 to 6 cardiac cycles according to the American Society of Echocardiography^13^, and digitally transferred online to a computer, and subsequently analyzed by an analyst blinded to the treatment groups.

Heart tissues from all studied groups of rats were analyzed histologically. Three micron thick sections were cut from formalin-fixed, paraffin embedded tissue of the heart and the sections were stained with Trichrome’s stain. The sections were studied under the optic microscope and photographed by a histopathologist.

### RNA extraction and cDNA synthesis

TRIzol reagent (Invitrogen^®^) and the High-Capacity cDNA reverse transcription kit (Applied Biosystems) were used to isolate total RNA from frozen tissues or treated cells and synthesize cDNA, respectively, according to the manufacturer’s instructions as described previously^14,15^.

### Quantification of mRNA expression by quantitative real-time polymerase chain reaction (real time-PCR)

Real time-PCR was used to quantify specific mRNA expression using the ABI Prism 7500 System (Applied Biosystems) as described previously^15,16^. Human and rat primer sequences and probes for procollagen III (pro III) and transforming growth factor beta 1 (TGF-β1), α-myocin heavy chain (α-MHC), β-myocin heavy chain (β-MHC), brain natriuretic peptide (BNP), Tumor necrosis factor-α (TNF-α), interleukin-6 (IL-6), BAX, P53, rat GAPDH and human β-actin are listed in Table 1. These primers were purchased from Integrated DNA technologies (IDT, Coralville, IA). The relative gene expression (i.e., ΔΔ CT) method was used to analyze the real time-PCR data, as described and explained previously^16^.

**Table 1:** Primers sequences used for RT- PCR reactions.

Gene	Forward primer	Reverse primer
*a-MHC*	GCCCTTTGACATTCGCACTG	GGTTTCAGCAATGACCTTGCC
*β-MHC*	TCACCAACAACCCCTACGATT	CTCCTCAGCGTCATCAATGGA
*β-actin*	CTGGCACCCAGCACAATG	GCCGATCCACACGGAGTACT
*Rat β-MHC*	CGCTCAGTCATGGCGGAT	GCCCCAAATGCAGCCAT
*Rat BNP*	CAGAAGCTGCTGGAGCTGATAAG	TGTAGGGCCTTGGTCCTTTG
*Pro III* (*Rat*)	CAGCTGGCCTTCCTCAGACT	TGCTGTTTTTGCAGTGGTATGTAA
*TGF-β1* (*Rat*)	ACCTGCAAGACCATCGACATG	CGAGCCTTAGTTTGGACAGGAT
*Rat IL-6*	GTCAACTCCATCTGCCCTTCA	GGCAGTGGCTGTCAACAT
*Rat TNF-α*	ACAAGGCTGCCCCGACTAT	CTCCTGGTATGAAGTGGCAAATC
*Rat P53*	CAGCTTTGAGGTTCGTGTTTGT	ATGCTCTTCTTTTTTGCGGAAA
*Rat Bax*	CCCACCAGCTCTGAACAGTTC	GTGTCTCCCCAGCCATCCT
*Rat GAPDH*	CAAGGTCATCCATGACAACTTTG	GGGCCATCCACAGTCTTCTG

### Preparation of microsomal proteins

Microsomal fractions were prepared by differential centrifugation of homogenized cardiac tissues as described previously^14^. Briefly, organs were washed in ice-cold potassium chloride (1.15%, w/v). Successively cut into pieces, and homogenized in ice-cold 0.25 M sucrose solution (17%, w/v). After homogenization, the tissues were separated by differential ultracentrifugation. The final pellet was re-suspended in cold sucrose and stored at −80 °C. The microsomal protein concentration was determined by the Lowry method using bovine serum albumin as a standard

### Separation of AA Metabolites by Liquid Chromatography–Electrospray Ionization–Mass Spectrometry

The incubation buffer (5 mM magnesium chloride hexahydrate dissolved in 0.1 M potassium phosphate buffer, pH 7.4) was used to incubate heart microsomes (1 mg protein/ml) at 37 °C in a shaking water bath (50 rpm) for 5 minutes as a pre-equilibration period. 1 μM NADPH was then added to initiate the reaction and a final concentration of 50 μM AA was incubated for 30 minutes. The reaction was terminated by the addition of 600 ml of ice-cold acetonitrile followed by the internal standard, 14(15)-EET-d11. Mid-chain HETEs metabolite were extracted with ethyl acetate, dried using speed vacuum (Savant, Farmingdale, NY) and analyzed using liquid chromatography–electrospray ionization mass spectrometry (LC–ESI–MS) (Waters Micromass ZQ 4000 spectrometer) method as described previously^17–19^.

### Western blot analysis

Western blot analysis was carried out under denaturing and reducing conditions using a previously described method^14,20,21^.

### In-gel digestion and LC–MS/MS analysis

Samples were prepared and electrophoresed as described in the aforementioned western blot method^14^. After electrophoresis, Coomassie Brilliant Blue was used to stain the SDS gel then the gel was destained overnight using de-staining solution (H2O: Methanol: Acetic acid, 50:40:10). The gel was then visualized using an LI-COR Odyssey gel scanner to quantify the intensity of protein content in each lane. Each lane on the gel was excised into 12 equal pieces after being de-stained using 100 mM NH_4_HCO_3_/acetonitrile (50:50). Each gel piece was subjected to in-gel tryptic digestion as previously described^22^. The final extracted peptides from the gel were suspended in 5% acetonitrile and 1% formic acid then analyzed on an LTQ Oribitrap XL, with the resulting data being searched against the *Rattus norvegicus* protein database using Proteome Discoverer 1.3 as previously described^23^. At least two high confidence peptides were used as a cut off for protein identification. With this criterion, more than 500 proteins were identified (Supplemental Tables 1–4). The protein’s extracted ion chromatogram (EIC) was used to compare the abundance between samples.

### Cell culture and treatments

Human cardiomyocyte RL-14 cells (American Type Cell Culture Patent Deposit Designation No. PTA-1499, Manassas, VA) and rat cardiomyocyte H9c2 cells, were maintained and grown as described previuosly^14,24–26^. Furthermore, both cell lines were used at passage range from 3 to 12.

### Effect of tested compounds on cell viability

The effect of 100 μM ISO with or without 0.25 μM 2 ME at 24 h on RL-14 cell viability in addition to the effect of 10 μM Ang-II with or without 0.25 μM 2 ME on H9c2 cell viability were determined by MTT and LDH assays as described previously^14,20,27^.

### Measurement of cell surface area

Relative changes in cell surface area, as an indicator for hypertrophy following treatments, were measured using phase contrast imaging. RL-14 cells were treated with 100 μM ISO with or without 0.25 μM 2 ME for 24 h. Also, H9c2 cells were incubated with 10 μM Ang-II with or without 0.25 μM 2 ME for 24 h. Thereafter, phase contrast images were taken with Zeiss Axio Observer Z1 inverted microscope using the 20x objective lens as described previously^14,28^.

### Determination of superoxide radical

RL-14 cells were treated with 100 μM ISO with or without 0.25 μM 2 ME for 24 h. Also, H9c2 cells were incubated treated with 10 μM Ang-II with or without 0.25 μM 2 ME for 24 h. Thereafter, superoxide radical was determined using 10 μM dihydroethidium (DHE). Fluorescence measurements at excitation/emission (545/575 nm) were read in a Synergy H1Hybrid Multi-Mode Microplate Readers (Bio-Tek Instruments, Winooski, VT, USA). (Instruments Inc., VT, USA).

### Determination of MAPKs signaling pathway

RL-14 cells were treated with 100 μM ISO with or without 0.25 μM 2 ME for 24 h. Also, H9c2 cells were incubated with 10 μM Ang-II with or without 0.25 μM 2 ME for 24 h. Thereafter, MAPKs protein phosphorylation was determined using the PhosphoTracer MAPK ELISA Kit (Abcam, Cambridge, UK). Fluorescence measurements at excitation/emission (545/575 nm) were read in a Synergy H1Hybrid Multi-Mode Microplate Readers (Bio-Tek Instruments, Winooski, VT, USA). Instruments Inc., VT, USA). Fluorescent data was normalized against total protein concentration and the expression of total protein from the same sample.

### Preparation of nuclear extract

RL-14 cells were treated with 100 μM ISO with or without 0.25 μM 2 ME for 24 h. Also, H9c2 cells were incubated with 10 μM Ang-II with or without 0.25 μM 2 ME for 24 h. Thereafter, nuclear extracts were prepared according to a previously described procedure for the measurement of NF-κB binding activity^14,29^.

### Determination of NF-κB binding activity

The NF-κB binding activity was detemined using the NF-κB Family EZ-TFA Transcription Factor Assay Chemiluminescent Kit (Millipore, Schwalbach/Ts., Germany, #70-660) according to the manufacturer’s protocol as described previously^14,30^.

### Statistical analysis

Data are presented as mean with SEM. Data were analyzed using One-way analysis of variance (ANOVA) followed by Tukey-Kramer multiple comparison tests. Analysis was performed using SigmaPlot^®^ for Windows (Systat Software, Inc, CA). A result was considered statistically significant when *p* < 0.05.

## Results

### Effect of 2 ME on AAC-induced cardiac hypertrophy in rats

**Figure 1:**
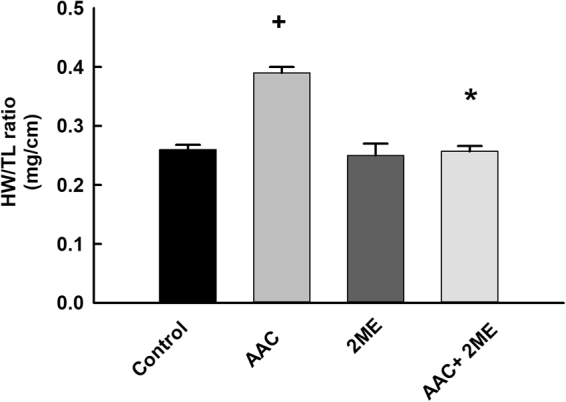
Effect of 2 ME on AAC-induced HW/TL ratio. Sham and AAC rats were treated with 2 ME (5 mg/kg/day) in the mini osmotic pump. Thereafter, HW/TL ratio (in mg per cm) was determined for each animal. The values represent mean ± SEM (n = 6). ^+^*P* < 0.05 compared to control. *P < 0.05 compared to AAC.

To investigate whether 2 ME confers cardioprotection against AAC-induced cardiac hypertrophy in rats, overall morphology was assessed *in vivo* via echocardiography and *ex vivo* by measuring heart weight-to-tibial length ratio (HW/TL). Echocardiography assessment of AAC rats showed thickening of the left ventricular wall as evidenced by an increase in LVPWs, LVPWd, IVSD, and IVSs in addition to a significant increase in LV mass (Table 2). Importantly, 2 ME treatment resulted in a substantial reduction in the AAC-mediated thickening of left ventricular morphology. Additionally, AAC significantly increased the HW/TL to 0.39 from the control level of 0.26, whereas treatment with 2 ME significantly inhibited the AAC-mediated increase in the HW/TL ratio to 0.257 (Fig. 1). Although the echocardiographic assessment revealed that 2 ME did not significantly influence heart rate or parameters of systolic function such as EF% and %FS in the AAC rats, 2 ME was able to significantly decrease the level of pressure gradient-increased by AAC (Table 2). 2 ME significantly prevented the increase in body weight in sham and AAC rats by approximately 38% and 30%, respectively in comparison to control (Table 2); however, treatment with 2-ME was not associated with any toxic adverse effects.

**Table 2:** Hemodynamic parameters in rats.

	Control	AAC	2 ME	AAC + 2 ME
BW (baseline)	200 ± 8	195 ± 6	190 + 10	189 ± 17
BW(after 5 weeks)	504 ± 19	508 ± 10	310 + 32^+^	358 ± 7^+^*
LV-Mass (mg)	1172 ± 52	1820 ± 160^+^	1137 + 235	1073 ± 55*
LVPWd (mm)	2.6 ± 0.1	3.73 ± 0.22^+^	2.6 ± 0.21	2.8 ± 0.1*
LVPWs (mm)	1.8 ± 0.06	2.6 ± 0.12^+^	1.8 ± 0.22	1.9 ± 0.17*
IVSd (mm)	1.8 ± 0.02	2.57 ± 0.12^+^	1.89 ± 0.18	1.9 ± 0.06*
IVSs (mm)	2.5 ± 0.1	3.44 ± 0.19^+^	2.64 ± 0.22	2.7 ± 0.1*
Heart Rate (bpm)	336 ± 17	371 ± 23	321 ± 31	316 ± 24
% EF	73 ± 2.9	77.4 ± 4.3	75.7 ± 2.8	78 ± 2.5
% FS	46.3 ± 1.5	43.3 ± 1.2	38.8 ± 4.4	44.8 ± 2.3
TEI	0.6 ± 0.04	0.75 ± 0.02	0.66 ± 0.08	0.72 ± 0.03
ME/A (ratio)	1.26 ± 0.15	1.23 ± 0.11	1.1 ± 0.08	1.3 ± 0.07
E/E′ (mm/sec)	25.4 ± 1.4	21 ± 3.1	25.7 ± 8.5	20.6 ± 1.9
A′/E′ (mm/sec)	1.2 ± 0.07	1.08 ± 0.12	1.16 ± 0.24	1.1 ± 0.15
E′/A′ (mm/sec)	0.8 ± 0.06	0.98 ± 0.11	0.9 ± 0.21	0.96 ± 0.11
S (mm/s)	45.5 ± 2.5	53 ± 1.9^+^	43 ± 1.7	44 ± 3.2*
PG (mmHG)	3.4 ± 0.3	5.8 ± 0.4^+^	4.4 ± 0.3	2.9 ± 0.3*

The experiment was replicated twice and the values represent mean ± SEM (n = 6). ^+^*P* < 0.05 compared to control. *P < 0.05 com*p*ared to AAC. BW, body weight, LV mass, left ventricular mass; LVPWs, left ventricular posterior wall, systole; LVPWd, left ventricular posterior wall, diastole; IVSs, intraventricular septum, systole; IVSd, intraventricular septum, diastole; Heart rate; EF, ejection fraction; FS, fractional shortening; TEI, Tei index = (isovolumic relaxation time + isovolumic contraction time)/ ejection time; E, A, wave velocity; E′, A′, tissue doppler wave; S, sytolic tissue movement PG, Pressure Gradient.

### Effect of 2 ME on AAC-induced fibrosis in rat cardiac tissues

**Figure 2:**
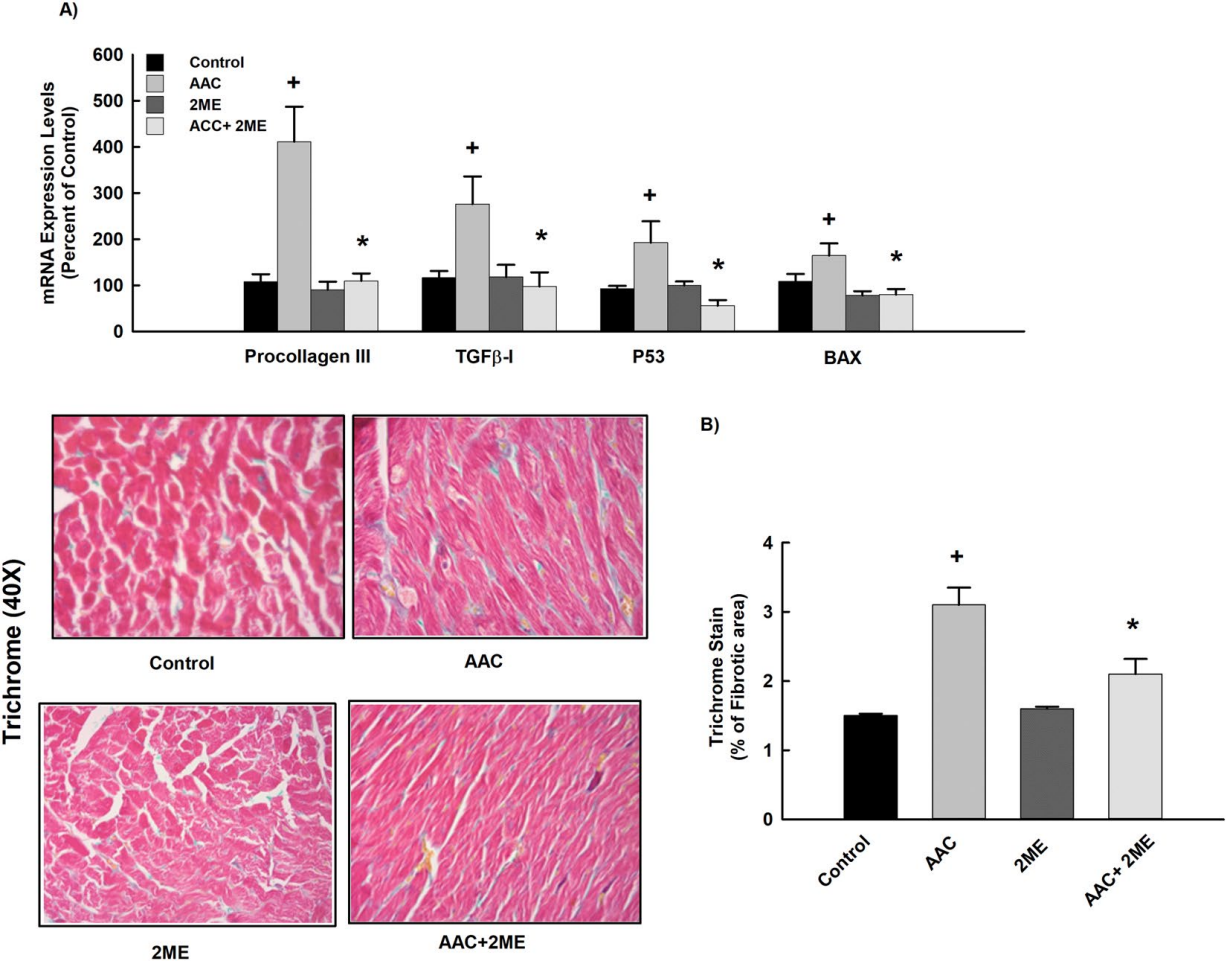
Effect of 2 ME on AAC-mediated induction of fibrotic and apoptotic markers. Sham and AAC rats were treated with 2 ME (5 mg/kg/day) in the mini osmotic pump. Thereafter, (**A**) the mRNA level of pro III, TGFβ-I, p53 and BAX were quantified using real time-PCR. (**B**) Fibrotic areas were determined using Trichrome’s stain (intense blue staining), quantified and expressed as % of fibrotic area. The experiment was replicated twice and the values represent mean ± SEM (n = 6). ^+^*P* < 0.05 compared to control. *P < 0.05 compared to AAC.

Chronic pressure overload is usually associated with excessive cardiac fibrosis and apoptosis which further burden left ventricular remodeling and affects myocardial compliance. Because of this, we investigated whether the protective effect of 2 ME against left ventricular remodeling is attributed to an inhibition of cardiac fibrosis and apoptosis induced by AAC. For this purpose, we measured the cardiac gene expression of the fibrotic and apoptotic markers, pro III, TGFβ-1, P53 and BAX relative to AAC rats using real-time PCR. Our results showed that AAC caused a significant induction of the fibrotic and apoptotic markers, pro-III, TGFβ-1, P53 and BAX by 400%, 250%, 200% and 170%, respectively in comparison to control (Fig. 2A). On the other hand, 2 ME treatment significantly inhibited the AAC-mediated induction of pro-III, TGFβ-1, P53 and BAX (Fig. 2A). Furthermore, no significant differences were observed between the control and the 2 ME treatment alone.

To further confirm the protective effect of 2 ME against AAC-induced fibrosis, cardiac sections were stained with Trichrome’s stain for the detection of fibrillar collagen and hence fibrosis (Fig. 2B). Microscopic view of the myocardial tissue showed a significant increase in the fibrotic area in the hearts of AAC rats by approximately 120% in comparison to control. Importantly, this parameter was significantly reduced by 2 ME treatment (Fig. 2B), demonstrating that 2 ME reduces the degree of fibrosis in rats with established cardiac hypertrophy.

### Effect of AAC and 2 ME on mid-chain HETEs level and the expression of CYP1B1, LOXs and COX-2 proteins in rats

**Figure 3:**
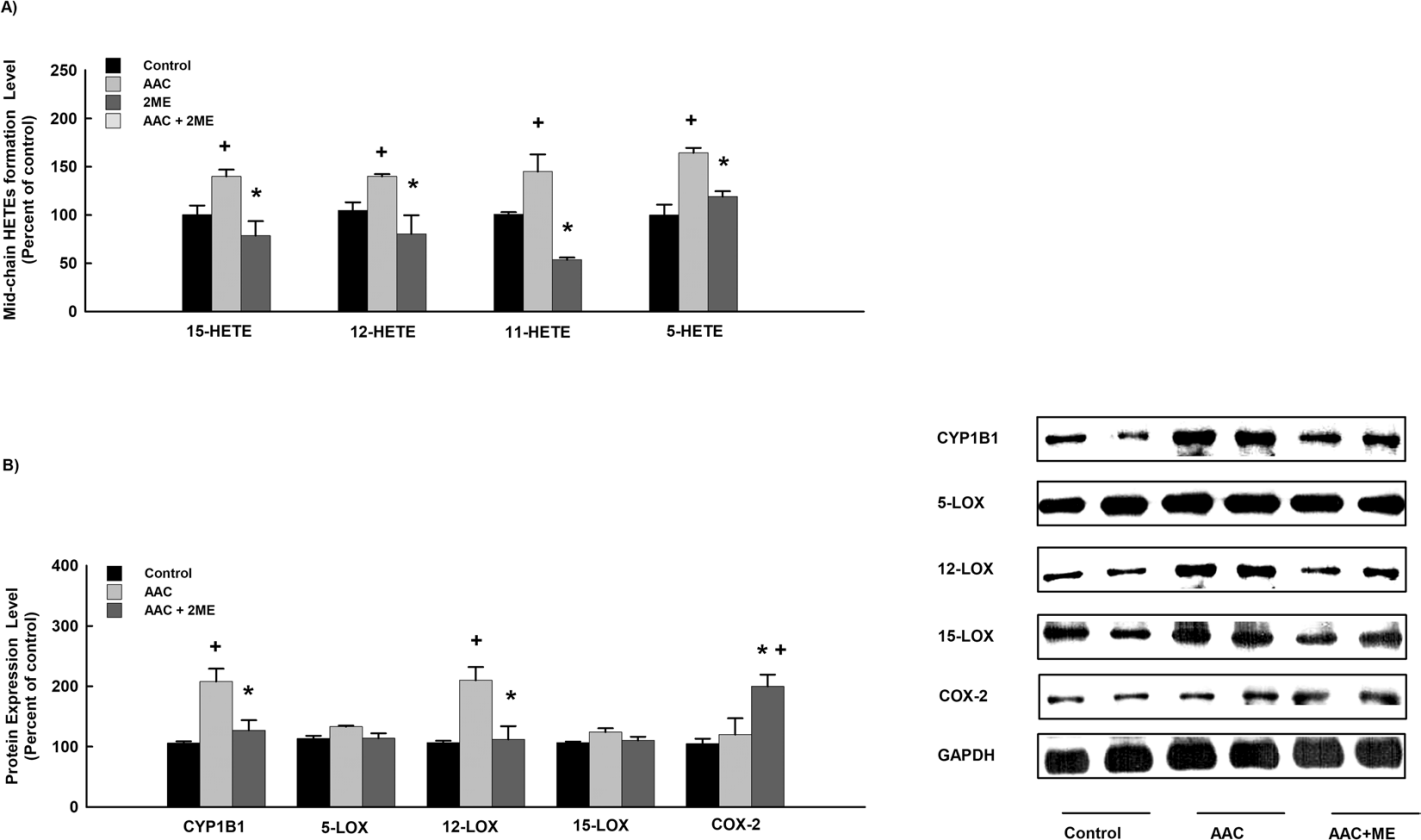
Effect of AAC and 2 ME on mid-chain HETE level and the expression of CYP1B1, LOXs and COX protein. Sham and AAC rats were treated with 2 ME (5 mg/kg/day) in the mini osmotic pump and then, (**A**) mid-chain HETE metabolites were measured using LC–ESI–MS. (**B**) CYP1B1, 5-LOX, 12-LOX, 15-LOX and COX-2 protein expression levels were determined by Western blot analysis. The experiment was replicated twice and the values represent mean ± SEM (n = 6). ^+^*P* < 0.05 compared to control. *P < 0.05 compared to AAC.

To determine the capacity of 2 ME to inhibit the formation of mid-chain HETEs altered by AAC, mid-chain HETE metabolites were determined using LC–ESI–MS. The net rate of 5-, 11-, 12-, and 15-HETE formation were significantly increased to 150%, 140%, 150% and 160%, respectively, in hypertrophied heart microsomes in comparison to control (Fig. 3A). On the other hand, treatment with 2 ME significantly reduced the increase in mid-chain HETEs formation to 90%, 85%, 60%, and 110% in comparison to hypertrophied heart (Fig. 3A). Furthermore, no significant differences were observed between the control and the 2 ME treatment alone (Supplementary Figure 15).

Since mid-chain HETEs was shown to be formed by CYP1B1 as well as LOX and degraded by COX-2 enzyme, we examined whether the decrease in the formation of mid-chain HETEs by 2 ME is due to the inhibition of LOXs and CYP and/or the activation of the COX-2 enzyme. For this purpose, CYP1B1, LOXs, and COX-2 protein expression levels were determined by Western blot analysis. Our results showed that AAC rats have demonstrated a significant increase in the expression of CYP1B1 and 12-LOX to 200% and 190%, respectively, in comparison to control. However, no significant changes were observed in the expression of COX-2, 5-LOX and 15-LOX between the control and the AAC group. Importantly, 2 ME significantly inhibited the expression of CYP1B1 and 12-LOX-induced by AAC to 110% and 95%, respectively in comparison to AAC group (Fig. 3B). On the other hand, treatment with 2 ME significantly increased the expression of in COX-2 to 190% in comparison to AAC group (Fig. 3B). Furthermore, no significant differences were observed between the control and the 2 ME treatment alone (Supplementary Figure 15).

### Differential protein expression profile in the AAC group and 2 ME treated rats

The proteins altered during cardiac hypertrophy in comparison to control and in the AAC rats treated with 2 ME in comparison to AAC group was determined using LC–MS/MS and have been listed in Table 3 along with their mean fold change ratios.

**Table 3:** Proteins altered during AAC and AAC + 2 ME.

Accession No.	Protein name	Mean fold change ratio-AAC vs C (*p* value)	Mean fold change ratio-2 ME + AAC vs AAC (*p* value)
Q64119	Myosin light polypeptide 6	0.44 (0.13)	4.24 (0.01)
P11507	Sarcoplasmic/endoplasmic reticulum calcium ATPase 2	0.55 (0.033)	1.44 (0.36)
P07335	Creatine kinase B-type	1.25 (0.33)	0.44 (0.035)
P85972	Vinculin	0.92 (0.063)	1.33 (0.044)
Q7TP54	Protein FAM65B	1.06 (0.75)	3.93 (0.005)
Q5XIB3	ADP-ribosylhydrolase-like 1	1.44 (0.008)	0.68 (0.23)
P20760	Ig gamma-2A chain C region	0.90 (0.84)	2.16 (0.0005)
P01835	Ig kappa chain C region, B allele	0.79 (0.56)	1.93 (0.009)
P11762	Galectin-1	2.58 (0.005)	1.01 (0.97)
O35303	Dynamin-1-like protein	0.46 (0.01)	5.77 (0.44)
Q9WTY9	P38 MAPK	0.34 (0.02)	—
P16036	Phosphate carrier protein, mitochondrial	0.67 (0.027)	1.15 (0.41)
Q64536	Pyruvate dehydrogenase kinase	0.88 (0.72)	0.46 (0.008)
P52873	Pyruvate carboxylase	0.8 (0.382)	0.69 (0.035)
P23965	Enoyl-CoA delta isomerase 1	0.48 (0.025)	0.97 (0.921)
P45953	Acyl-CoA dehydrogenase	0.59 (0.025)	1.44 (0.15)
P14604	Enoyl-CoA hydratase	0.60 (0.039)	1.01 (0.97)
Q64591	2,4-Dienoyl-CoA reductase	0.39 (0.145)	2.76 (0.038)
Q5XIT9	Methylcrotonoyl-CoA carboxylase beta chain	0.85 (0.086)	1.63 (0.041)
P35738	2-Oxoisovalerate dehydrogenase	0.63 (0.028)	2.61 (0.14)
Q920L2	Succinate dehydrogenase	0.56 (0.022)	1.55 (0.013)
P80254	D-dopachrome decarboxylase	2.65 (0.039)	0.63 (0.21)
P29419	ATP synthase subunit e	1.74 (0.12)	0.54 (0.037)
P35434	ATP synthase subunit delta	1.44 (0.008)	1.27 (0.63)
Q6UPE1	Electron transfer flavoprotein-ubiquinone oxidoreductase	0.55 (0.033)	1.26 (0.46)
P04906	Glutathione S-transferase P	0.80 (0.68)	1.95 (0.009)
P08010	Glutathione S-transferase Mu	0.81 (0.29)	1.85 (0.047)
Q66HF1	NADH-ubiquinone oxidoreductase	0.52 (0.029)	0.86 (0.59)
P19132	Ferritin heavy chain	0.66 (0.24)	2.84 (0.02)
P67779	Prohibitin-1	0.408 (0.006)	1.45 (0.44)
Q5XIH7	Prohibitin-2	0.40 (0.105)	1.87 (0.04)

Sham and AAC rats were treated with 2 ME (5 mg/kg/day) in mini osmotic pump and then, proteomic was determined using LC-MS/MS. Duplicate reactions were performed for each experiment and the values represent mean ± SEM (n = 4).

The cardiac remodeling induced by AAC was associated with a significant increase in the expression of the hypertrophic protein, ADP-ribosylhydrolase-like 1 by 1.44 fold of change in comparison to control. Consistent with echocardiography, the protective effect of 2 ME against AAC induced cardiac hypertrophy was associated with a substantial increase in the expression of cardioprotective proteins, protein FAM65B and vinculin, and a significant decrease in the creatine kinase B-type protein, a biomarker of heart disease, by 3.9, 1.33 and 0.44 fold of change, respectively, in comparison to AAC group (Table 3). Muscle contraction protein system, sarcoplasmic/endoplasmic reticulum calcium ATPase 2, was significantly decreased in the AAC group by 0.55 fold of change in comparison to control. However, 2 ME treated rats showed an exclusive increase in the protein expression of myosin light polypeptide 6 and myosin light chain 3 by 4.2 and 1.9 fold of change in comparison to AAC group (Table 3).

The profile of inflammatory and fibrotic proteins altered in response to cardiac hypertrophy induced by AAC showed a significant increase in the expression of the fibrotic protein, galectin-1, by 2.5 fold of change in comparison to control. On the other hand, anti-inflammatory and anti-fibrotic proteins, Ig gamma-2A chain C region and Ig kappa chain C region, were significantly decreased in AAC rats treated with 2 ME by 2.1 and 1.9 fold of change, respectively, in comparison to AAC group (Table 3). Contrary to fibrotic proteins, proteins related to apoptotic pathway, dynamin-1-like protein, P38 MAPK and mitochondrial phosphate carrier protein, were significantly reduced in the AAC group by 0.4, 0.3 and 0.6 fold of change, respectively, in comparison to control. Moreover, treatment of rats with 2 ME further decreased the AAC-mediated inhibition of P38 MAPK (Table 3).

Our data showed that proteins under the category of cardiac metabolism were the most affected in the AAC rats and AAC rats treated with 2 ME. Proteins involved in fatty acid and branched-chain amino acid oxidation, enoyl-CoA delta isomerase 1, acyl-CoA dehydrogenase, enoyl-CoA hydratase, electron transfer flavoprotein-ubiquinone oxidoreductase and 2-oxoisovalerate dehydrogenase, were significantly decreased in the AAC rats by 0.48, 0.59, 0.6, 0.55 and 0.63 fold of change, respectively, in comparison to control. Although 2 ME did not significantly affect proteins involved in fatty acid and branched-chain amino acid oxidation altered by AAC, 2 ME significantly decreased those responsible for the suppression of glucose oxidation, pyruvate dehydrogenase kinase, and pyruvate carboxylase, by 0.4 and 0.6 fold of change, respectively, in comparison to AAC group (Table 2).

Antioxidant protein expression, NADH-ubiquinone oxidoreductase, and prohibitin-1 were significantly decreased in the AAC group by 0.5 and 0.4 fold, respectively, in comparison to the control. On the other hand, 2 ME significantly increased the expression of antioxidant proteins such as, glutathione S-transferase P, glutathione S-transferase Mu, ferritin heavy chain and prohibitin-2, by a 1.9, 1.8, 2.8 and 1.8 fold of change, respectively, in comparison to the AAC group (Table 3).

### Effect of AAC and 2 ME on MAPK signaling pathway

**Figure 4:**
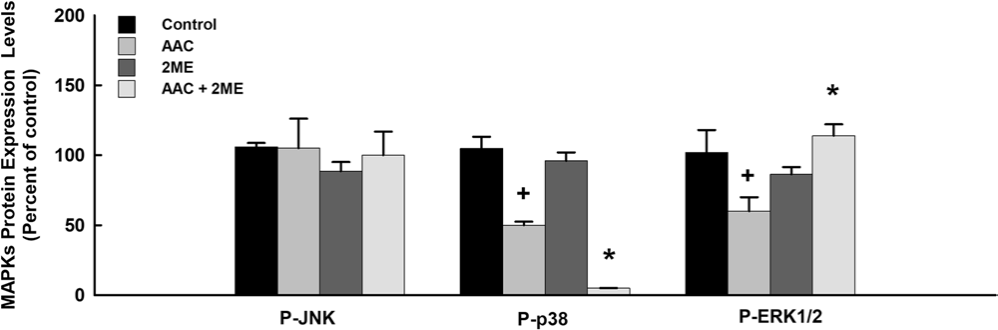
Effect of 2 ME and AAC on MAPK signaling pathway. Sham and AAC rats were treated with 2 ME (5 mg/kg/day) in the mini osmotic pump. Then, the MAPKs protein phosphorylation, P-JNK, P-p38 and P-ERK1/2 was determined. The values represent mean ± SEM (n = 6). ^+^*p* < 0.05 compared to control. *p < 0.05 compared to AAC.

ACC rats have demonstrated a significant decrease in the expression of phosphorylated p38 and ERK1/2 by approximately 50% and 40%, respectively, in comparison to the control (Fig. 4). However, no significant changes were observed in the expression of phosphorylated JNK between the control and the AAC group. Although treatment of rats with 2 ME further decreased the AAC-mediated inhibition of phosphorylated p38, 2 ME significantly normalized the AAC-mediated effect on the phosphorylated ERK1/2 indicating a crucial role of the MAPK signaling pathway in the protective effect of 2 ME against AAC induced left ventricular hypertrophy (Fig. 4). No significant differences were observed between the control and the 2 ME treatment alone

### Effect of 2 ME on ISO-mediated cellular hypertrophy

**Figure 5:**
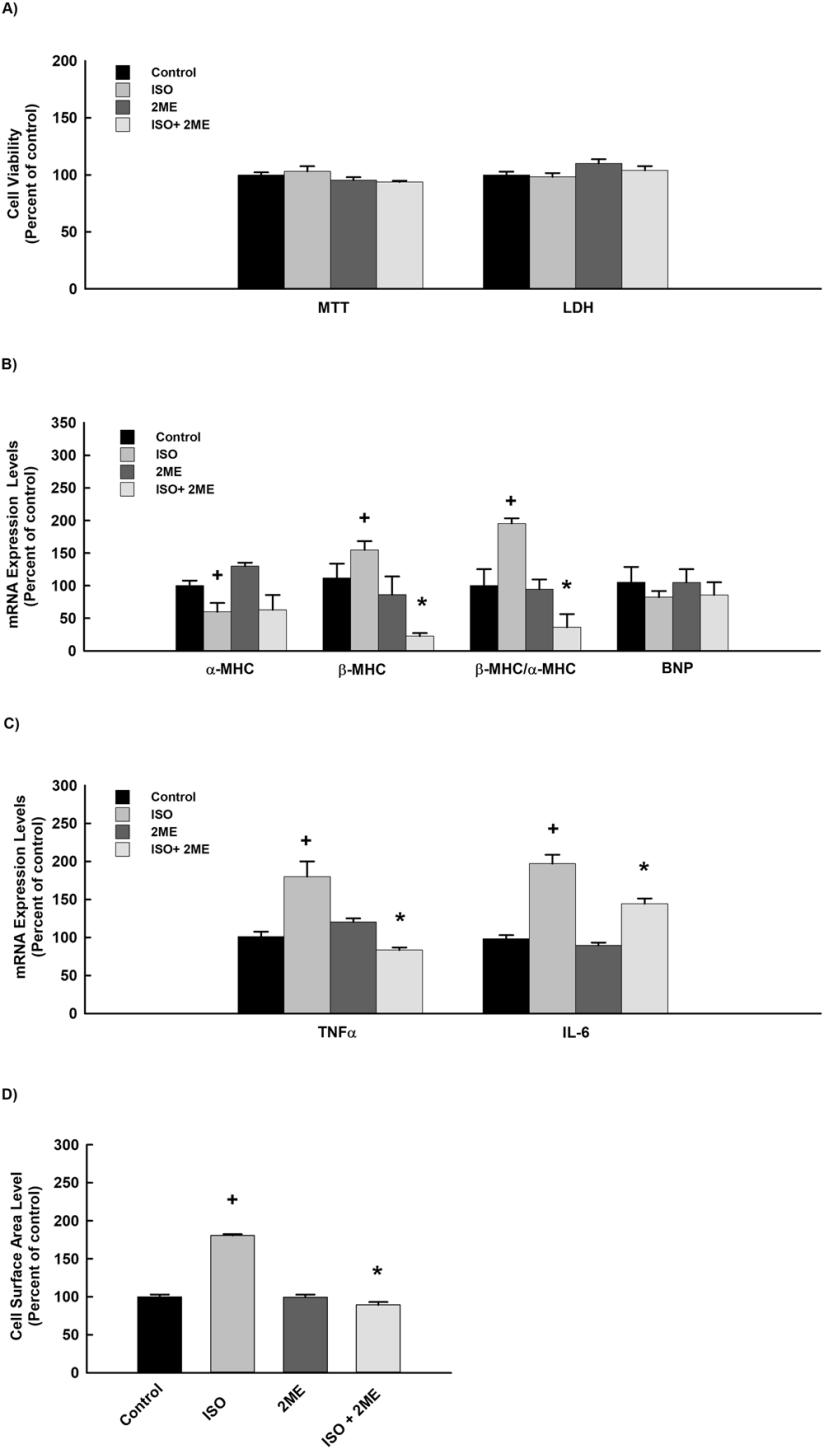
Effect of ISO and 2 ME on RL-14 cells viability, hypertrophic genes, and cell surface area. RL-14 cells were exposed to 100 μM ISO in the presence and absence of 0.25 μM 2 ME for 24 h. Thereafter, (**A**) RL-14 cell viability was determined using MTT and LDH assays. (**B**) The mRNA level of α-MHC and β-MHC was quantified using real time-PCR. (**C**) Cell surface area was analyzed by phase contrast imaging. The values represent mean ± SEM (n = 6). ^+^*P* < 0.05 compared to control. *P < 0.05 compared to ISO.

In order to investigate whether 2 ME has a direct antihypertrophic effect in the cardiac cells in a manner similar to *in vivo*, we examined the ability of 2 ME to inhibit cellular hypertrophy induced by ISO as cardiac hypertrophy induced by AAC is not possible in cells. Initially, we have demonstrated that treatment of RL-14 cells with 100 μM ISO with or without 0.25 μM 2 ME for 24 h did not significantly affect RL-14 cell viability using MTT and LDH assays (Fig. 5A). Figure 5B and C show that ISO alone caused a significant inhibition of α-MHC gene expression by about 50% and a significant induction of β-MHC, TNF-α and IL-6 genes expression by approximately 150%, 180% and 200%, respectively in comparison to the control. Although treatment of the cells with 2 ME did not significantly alter the ISO-mediated inhibition of α-MHC gene expression, 2 ME significantly inhibited ISO-induced β-MHC, β-MHC/α-MHC ratio, TNF-α and IL-6 genes expression by about 70%, 50%, 45% and 30%, respectively in comparison to the ISO treatment (Fig. 5B and C). No significant changes were observed with the expression level of BNP (Fig. 5B). The effect of 2 ME on ISO-induced cellular hypertrophy was further confirmed by the ability of 2 ME to completely restore the ISO-mediated increase in cell surface area (Fig. 5D).

### Effect of 2 ME on ISO-mediated effect on superoxide radical, MAPK and NF-κB signaling pathways

**Figure 6:**
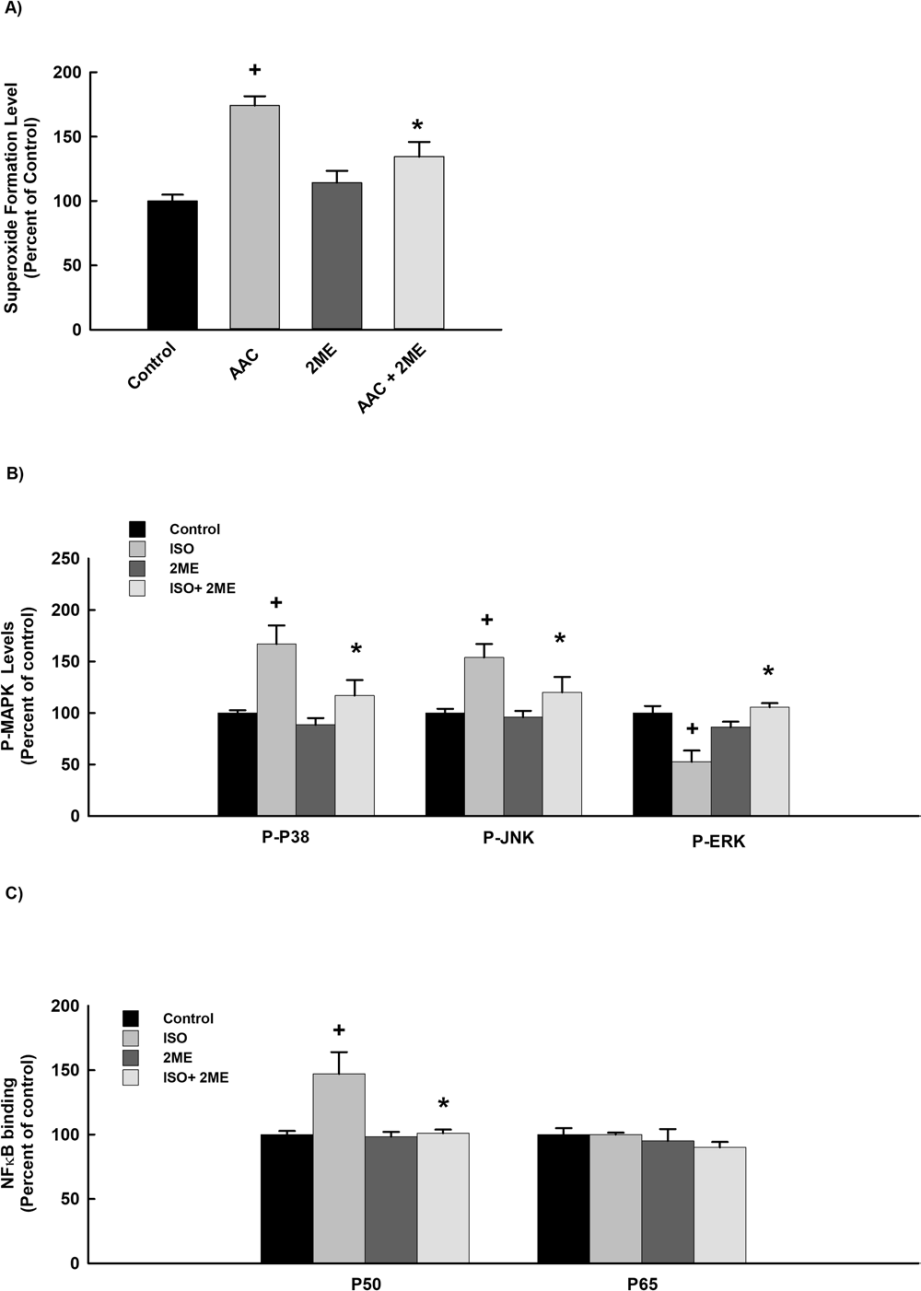
Effect of 2 ME on ISO-mediated effect on superoxide radical, MAPK and NF-κB signaling pathways. RL-14 cells were treated for 24 h with 100 µM ISO in the presence and absence of 0.25 μM 2 ME. Thereafter, (**A**) Superoxide anion was determined using DHE assay. (**B**) MAPKs protein phosphorylation was determined in cytoplasmic protein extracts using PhosphoTracer Elisa Kit (Abcam, Cambridge, UK). (**C**) NF-κB binding activity was determined using commercially available kit. The values represent mean ± SEM (n = 6). ^+^*P* < 0.05 compared to control. *P < 0.05 compared to ISO.

Figure 6 shows that incubation of the cells with 100 µM of ISO significantly increased superoxide radical formation, phosphorylation of p38 and JNK in addition to the binding activity level of NF-κB P50 by approximately 180%, 160%, 150%, and 145%, respectively, whereas it inhibited the phosphorylation of ERK1/2 by approximately 50% in comparison to the control. No significant changes were observed with the activity level of NF-κB P65 (Fig. 6C). Importantly, treatment with 2 ME significantly normalized the ISO-mediated effect on the superoxide radical, MAPK and NF-κB signaling pathways (Fig. 6) suggesting a crucial role of the aforementioned pathways in the protective effect of 2 ME against ISO induced RL-14 cellular hypertrophy.

### Effect of 2 ME on Ang II-mediated cellular hypertrophy in H9c2 cells

**Figure 7:**
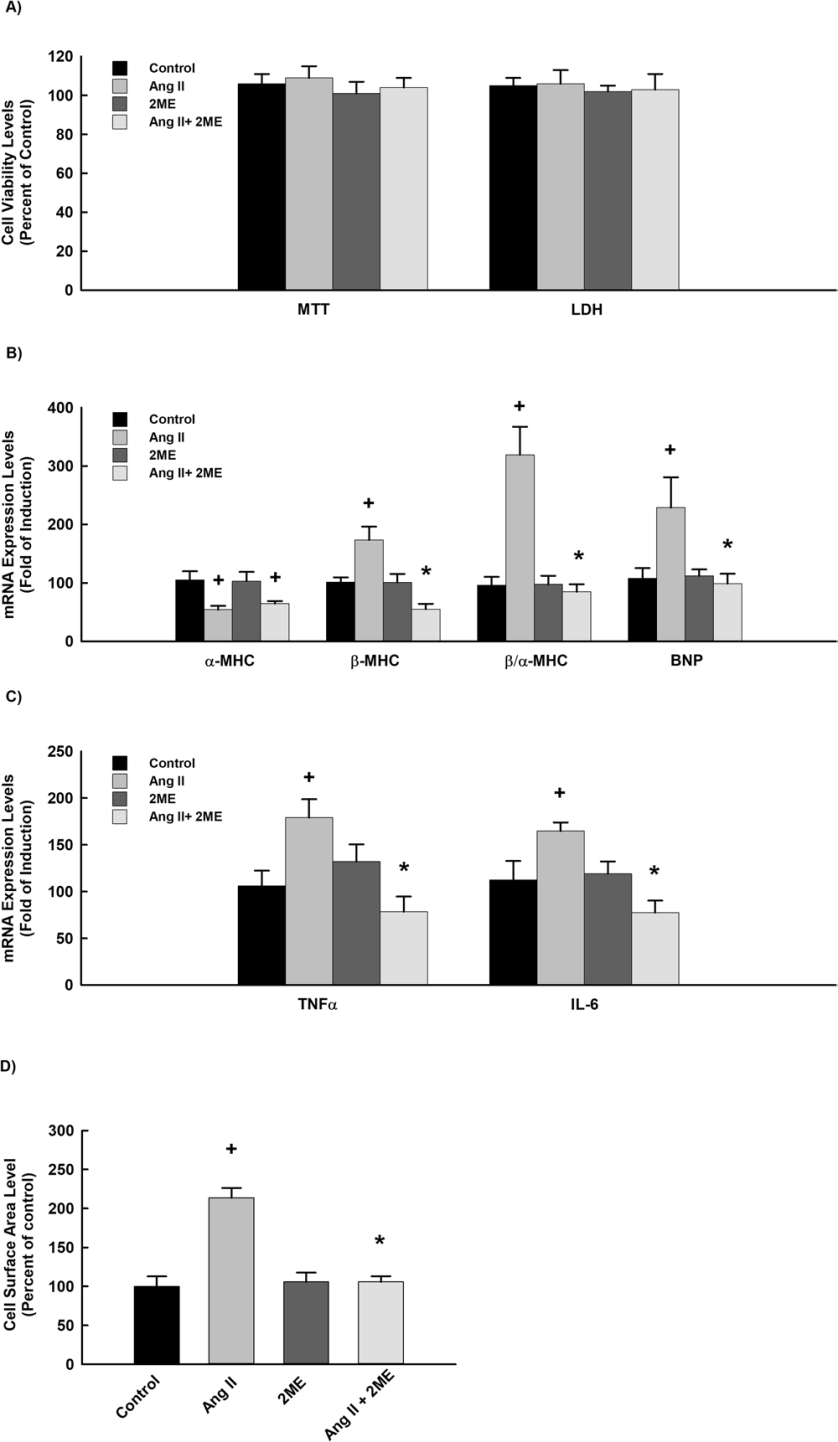
Effect of Ang II and 2 ME2 ME on RL-14 cells viability and hypertrophic genes. H9c2 cells were exposed to 10 μM Ang II in the presence and absence of 0.25 μM 2 ME for 24. Thereafter, (**A**) H9c2 cell viability was determined using MTT and LDH assays. (**B**) The mRNA level of BNP, β-MHC, TNF-α and IL-6 was quantified using real time-PCR. The values represent mean ± SEM (n = 6). ^+^*P* < 0.05 compared to control. *P < 0.05 compared to Ang II.

**Figure 8:**
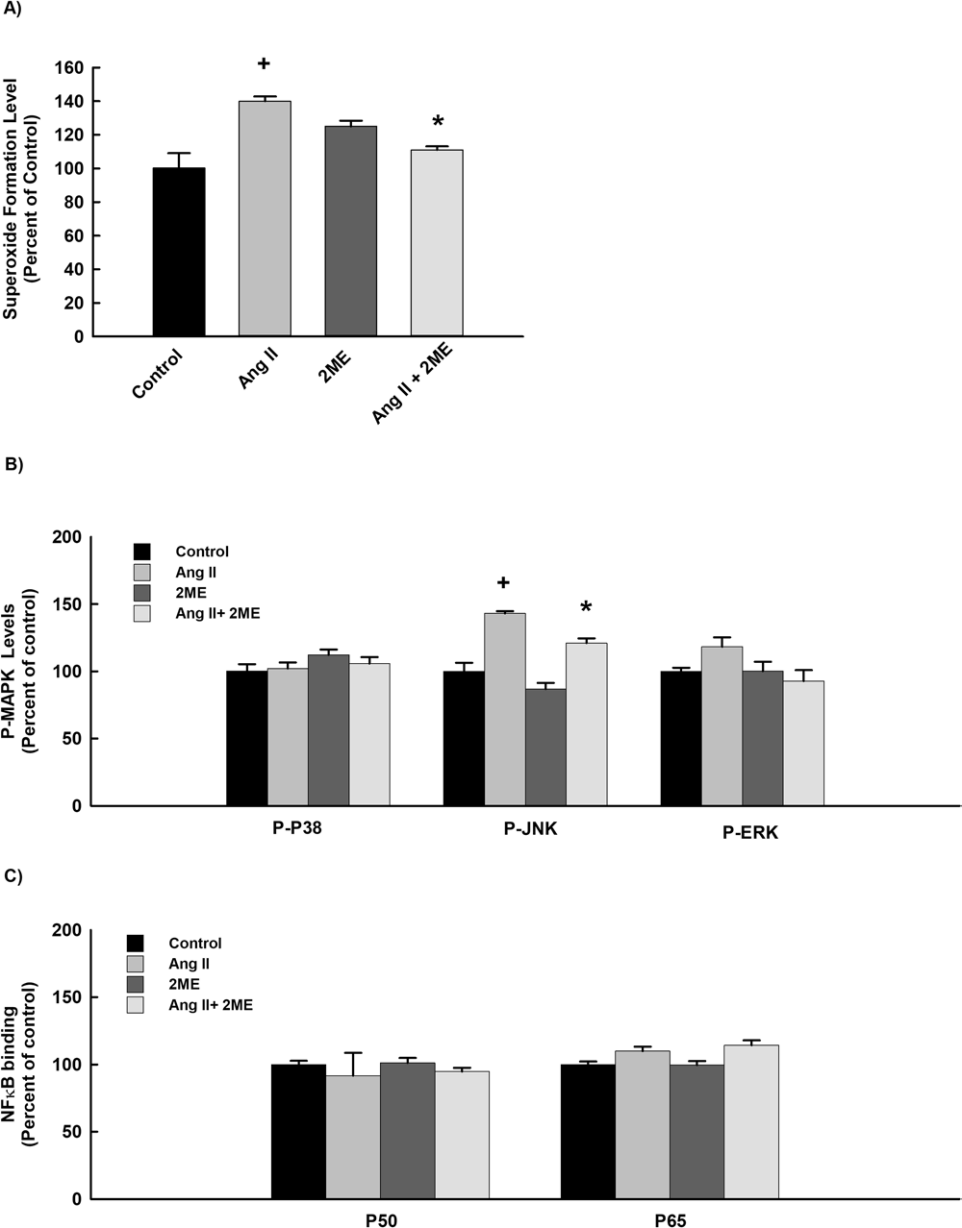
Effect of 2 ME on Ang II-mediated effect on superoxide radical, MAPK and NF-κB signaling pathways. H9c2 cells were treated for 24 h with 10 µM Ang II in the presence and absence of 0.25 μM 2 ME2 ME. Thereafter, (**A**) Superoxide anion was determined using DHE assay. (**B**) MAPKs protein phosphorylation was determined in cytoplasmic protein extracts using PhosphoTracer Elisa Kit (Abcam, Cambridge, UK). (**C**) NF-κB binding activity was determined using commercially available kit. The values represent mean ± SEM (n = 6). ^+^*P* < 0.05 compared to control. *P < 0.05 compared to Ang II.

To further validate the protective and the direct antihypertrophic effect of 2 ME, we examined the ability of 2 ME to inhibit cellular hypertrophy induced by Ang II in H9c2 cells. Initially, we have demonstrated that treatment of H9c2 cells with 10 μM Ang II with or without 0.25 μM 2 ME for 24 h did not significantly affect H9c2 cell viability using MTT and LDH assays (Fig. 7A). Figure 7B and C show that Ang II alone caused a significant inhibition of α-MHC gene expression by about 50% and a significant induction of β-MHC, β-MHC/α-MHC ratio, BNP, TNF-α and IL-6 genes expression by approximately 200%, 300%, 160%, 150% and 140%, respectively in comparison to the control. On the other hand, 2 ME significantly inhibited Ang II-mediated induction of the aforementioned genes (Fig. 7B and C). The effect of 2 ME on Ang II-induced cellular hypertrophy was further confirmed by the ability of 2 ME to completely restore the Ang II-mediated increase in cell surface area (Fig. 7D). Mechanistically, 2 ME significantly inhibited superoxides formation and the phosphorylation of JNK-induced by Ang II. No significant changes were observed with the phosphorylated p38 and ERK1/2 in addition to the P50 and P65 NF-κB binding activities (Fig. 8).

## Discussion

2 ME is a naturally occurring metabolite resulting from the hydroxylation of estradiol to catechol estradiol by CYP1B1, followed by the methylation of catechol estradiol by COMT. 2-ME has been shown to protect the heart and blood vessels from pathological processes, particularly those involving vascular smooth muscle cells and cardiac fibroblasts migration and proliferation^31^. However, the role of 2 ME in the treatment of cardiac hypertrophy is yet unknown^32^. Therefore, the major impetus for the present study was to determine the effect, if any, of 2 ME on cardiac hypertrophy induced by the AAC model and explore the mechanism(s) involved.

Initially, AAC was used as a model of cardiac hypertrophy because it is more clinically relevant and similar to the human form of the disease since the hypertrophy is developed over a relatively longer period of time^33^. Although the induction of cardiac hypertrophy surgically using AAC model was associated with an increase in the left ventricular wall thickness in addition to the presence of fibrosis, systolic and diastolic functions were not significantly changed. This is not surprising since significant cardiac dysfunctions occur within 6–10 weeks in the AAC model^33^. Furthermore, the presence of fibrosis has been reported in the hypertrophic heart with normal cardiac function^34–36^. The delivery of 2 ME by osmotic pumps at 5 mg/kg/day used in the current study were selected based on previous studies and comparable with a dose of 25 mg/kg/day given daily gavage in the absence of toxicity^37,38^.

The first novel finding of the present study was that 2 ME exerts protective effects against ventricular hypertrophy induced by AAC surgery as evident by the ability of 2 ME to restore the changes of all cardiac hypertrophic and fibrotic parameters. Supporting this view was the finding that 2 ME diminishes hypertension and associated coronary hypertrophy in ovariectomized spontaneously hypertensive rats and prevents DOCA-salt–induced hypertension in male rats^39,40^. Furthermore, 2 ME has been shown to inhibit cardiac fibroblast development and aortic smooth muscle cell growth^41^. Although our result suggest a direct antihypertrophic effect of 2 ME against AAC-induced cardiac hypertrophy, the effect of 2 ME on blood pressure could potentially contribute to the changes in cardiac hypertrophy as AAC is known to induce cardiac hypertrophy through the increase in blood pressure. Also, 2 ME was able to significantly decrease the level of pressure gradient-increased by AAC.

The interesting element revealed by this study was the unique inhibition of CYP1B1 and its associated cardiotoxic mid-chain HETE metabolites by 2 ME in AAC cardiac tissues suggesting a CYP1B1/mid-chain HETE-dependent mechanism. The importance of this finding has been inspired by the fact that CYP1B1 cuts both ways as it does not only catalyze the formation of cardiotoxic metabolites but it also metabolizes substrates, like estradiol, into 2 ME which could exert cardioprotective effects. Therefore, repurposing 2 ME to modulate CYP1B1 mediated AA metabolism could prove to be a more selective and effective strategy, comparable to its conventional inhibitor, TMS. This is supported by the previous finding demonstrating that TMS enhances Ang II-induced rise in systolic blood pressure in Cyp1b1_(**+**/**+**)_ female mice. Unlike TMS, 2 ME protects against Ang II-induced hypertension and oxidative stress in Cyp1b1_(−/−)_ female mice^9^. Being that 2 ME has few or no feminizing effects, it could also be used to treat cardiovascular disease in males^12^.

The mechanism(s) by which 2 ME exerts its cardioprotective effects seems to be independent of the classical genomic estrogen receptor. However, the exact mechanism is yet unknown and unsuspected. Therefore, the second objective of the current study is to elucidate the mechanism(s) by which 2 ME exerts its cardioprotective effects. For this purpose, the large-scale analysis of proteins, proteomics, was performed using Label-free quantification (LFQ) method. Although LFQ method quantifies proteins at a higher dynamic range and it gives a better view of proteins with higher fold changes, LFQ suffers from lower accuracy as a result of experimental variation and lower signal to noise ratio in comparison to higher accuracy method using stable isotope labeling^42^.

The present study depicts for the first time a comprehensive list of the proteins involved in the protective effect of 2 ME against cardiac hypertrophy induced by AAC. Initially, our proteomic data has confirmed the cardiac hypertrophy induced in response to AAC. This is because AAC group has shown a significant increase in the expression of the hypertrophic protein, ADP-ribosylhydrolase-like 1^43^, in addition to the fibrotic protein, Galectin-1^44^. Consistent with echocardiography, the antihypertrophic effect of 2 ME was accompanied with a substantial increase in the expression of cardioprotective proteins, FAM65B and Vinculin^45,46^. Furthermore, the anti-fibrotic effect of 2 ME against AAC induced cardiac fibrosis was associated with an exclusive increase in the expression of anti-inflammatory and anti-fibrotic proteins. This is not surprising since 2-ME has been shown to inhibit serum-induced proliferation and collagen synthesis in rat cardiac fibroblasts^47^.

Hypertrophy and apoptosis both propel the heart towards severe dilation and heart failure. Hypertrophic stress signals impact cardiomyocyte apoptosis at different levels, the consequence of which is cell survival promotion^48^. Consistent with this notion, our proteomic data showed that pro-apoptotic proteins, like dynamin-1-like protein and P38 MAPK were significantly reduced in the hypertrophic cardiac tissues. Of particular interest, 2 ME seems to have a salutary effect by supporting cell survival through a massive inhibition of P38 MAPK and the increase in the expression of ERK1/2. This may explain, at least in part, the protective effect of 2 ME since heart failure results from disturbing the balance between cell survival and apoptotic signals^49^. Furthermore, 2 ME has been shown previously to increase the activity of ERK1/2^50^ *in vitro* probably through a novel 7-transmembrane G-protein coupled receptor, GPR30^51^.

The heart is the most metabolically demanding organ in the body, acting as an omnivore using a variety of energy substrates to continually generate ATP^52^. Upon analysis of protein mapping, it is feasible to determine that the most predominantly affected proteins are those involved in various stages of the cardiac metabolic machinery. Hypertrophied cardiac tissues in the present study were associated with a substantial decrease in the protein expression of many enzymes involved in fatty acid oxidation. Our findings are typically consistent with the previous reports demonstrating an inhibition of fatty acid oxidation during cardiac hypertrophy^52,53^. Of particular interest, the anti-hypertrophic effect of 2 ME was associated with an inhibition of pyruvate dehydrogenase kinase which may increase the reliance of myocardium on glucose as a source of energy at expense of fatty acid oxidation. This particular novel finding is of great importance since a plethora of studies have shown that increased ATP supplied from glucose oxidation constitutes a bona fide therapeutic device in the treatment of cardiac hypertrophy and heart failure^54,55^.

Overweight and obesity increase risks for cardiovascular disease and are associated with many cardiac implications such as hypertension, heart failure, and sudden death^56^. In the current study, treatment with 2-ME significantly reduced the body weight in both sham and AAC rats. In agreement with our results, weight loss was observed previously, but this was disappeared rapidly with cessation of 2 ME^57^. Furthermore, 2 ME was shown to exert cardioprotective effects in part via the reduction of body weight^56^. Since 2 ME treatment was not associated with any sign of toxicity, we speculate that the weight reduction may be due to an indirect increase in proteins involved in glucose oxidation. In addition, 2 ME has been reported to improve glucose tolerance via increasing muscle consumption of glucose and reduction of fat and cholesterol levels which subsequently prevents weight gain^58,59^. 2-Hydroxyestradiol, a 2 ME’s precursor, was shown to reduce weight gain in obese ZSF1 rats at least in part by suppressing appetite^60^. Also, the previous finding has prompted us to investigate the effect of 2 ME on diabetes- and obesity-induced cardiomyopathy.

A wealth of information suggests the involvement of oxidative stress in the pathogenesis of cardiac hypertrophy which stresses the importance of unravelling the factors involved in providing protection against noxious ROS^61^. In this context, we have demonstrated that AAC significantly inhibited the protein expression of many antioxidant enzymes such as NADH-ubiquinone oxidoreductase. On the other hand, the beneficial effect of 2 ME in the AAC-induced cardiac hypertrophy may be partly due to an increase in the protein expression of glutathione S-transferase and ferritin heavy chain. Our results are in agreement with the previous observation showing that 2 ME is a potent antioxidant^62^ and displayed antihypertensive effect in spontaneously hypertensive rats, primarily by decreasing the formation of superoxide anion^39^.

In light of the information described above, our results suggest a direct evidence for the protective effect of 2 ME against pressure-overload-induced left ventricular hypertrophy. This raises the question of whether or not 2 ME has a direct antihypertrophic effect in the cardiac cells. For this purpose, we examined the ability of 2 ME to inhibit cellular hypertrophy induced by ISO using RL-14 cell line. ISO was used because it mimics the persistent adrenergic stimulation during maladaptive cardiac hypertrophy^63–65^ whereas, RL-14 cells were shown to express hypertrophic markers at comparable level to human primary cardiomyocytes^26^. H9C2 cells and Ang II were used to further validate the protective and the direct antihypertrophic effect of 2 ME.

Treatment of RL-14 cells and H9c2 cells with 2 ME significantly reduced the ISO- and Ang II-mediated cellular hypertrophy as evidenced by a decrease in the expression of hypertrophic markers and cell surface area therefore suggesting a direct antihypertrophic effect of 2 ME. Mechanistically, the protective effect of 2 ME was associated with decreasing the generation of superoxide through MAPK and NF-κB signaling pathways.

To reiterate, our results may shed light on the role of CYP1B1 and its associated mid-chain HETEs metabolite in the development of cardiac hypertrophy and suggest that CYP1B1 could be used a novel target in the treatment of pressure overload-induced cardiac hypertrophy. Such observation may provide the potential of repurposing 2 ME as a selective CYP1B1 inhibitor for the treatment of heart failure.

## Conclusion

The present work shows a strong evidence that 2 ME protects against left ventricular hypertrophy using the AAC model and *in vitro* in the cardiac cell line.

## Electronic supplementary material

Supplementary Figures

Supplementary Tables

## References

[1] Maayah ZH, El-Kadi AO (2016). The role of mid-chain hydroxyeicosatetraenoic acids in the pathogenesis of hypertension and cardiac hypertrophy. Archives of toxicology.

[2] Korashy HM, El-Kadi AO (2006). The role of aryl hydrocarbon receptor in the pathogenesis of cardiovascular diseases. Drug metabolism reviews.

[3] Malik KU (2012). Contribution of cytochrome P450 1B1 to hypertension and associated pathophysiology: a novel target for antihypertensive agents. Prostaglandins & other lipid mediators.

[4] Maayah ZH, El-Kadi AO (2016). 5-, 12- and 15-Hydroxyeicosatetraenoic acids induce cellular hypertrophy in the human ventricular cardiomyocyte, RL-14 cell line, through MAPK- and NF-kappaB-dependent mechanism. Archives of toxicology.

[5] Morgan ET (2001). Regulation of cytochrome p450 by inflammatory mediators: why and how?. Drug metabolism and disposition: the biological fate of chemicals.

[6] Wen Y (2001). Overexpression of 12-lipoxygenase causes cardiac fibroblast cell growth. Circulation research.

[7] Wallukat G, Morwinski R, Kuhn H (1994). Modulation of the beta-adrenergic response of cardiomyocytes by specific lipoxygenase products involves their incorporation into phosphatidylinositol and activation of protein kinase C. The Journal of biological chemistry.

[8] Kayama Y (2009). Cardiac 12/15 lipoxygenase-induced inflammation is involved in heart failure. The Journal of experimental medicine.

[9] Jennings BL (2014). Estrogen metabolism by cytochrome P450 1B1 modulates the hypertensive effect of angiotensin II in female mice. Hypertension.

[10] Dawling S, Roodi N, Parl FF (2003). Methoxyestrogens exert feedback inhibition on cytochrome P450 1A1 and 1B1. Cancer research.

[11] Lavigne JA (2001). The effects of catechol-O-methyltransferase inhibition on estrogen metabolite and oxidative DNA damage levels in estradiol-treated MCF-7 cells. Cancer research.

[12] Dantas AP, Sandberg K (2006). Does 2-methoxyestradiol represent the new and improved hormone replacement therapy for atherosclerosis?. Circulation research.

[13] Maayah ZH (2016). CYP1B1 inhibition attenuates doxorubicin-induced cardiotoxicity through a mid-chain HETEs-dependent mechanism. Pharmacological research.

[14] Maayah ZH (2017). The role of cytochrome P450 1B1 and its associated mid-chain hydroxyeicosatetraenoic acid metabolites in the development of cardiac hypertrophy induced by isoproterenol. Molecular and cellular biochemistry.

[15] Maayah ZH, Ansari MA, El Gendy MA, Al-Arifi MN, Korashy HM (2014). Development of cardiac hypertrophy by sunitinib *in vivo* and *in vitro* rat cardiomyocytes is influenced by the aryl hydrocarbon receptor signaling pathway. Archives of toxicology.

[16] Livak KJ, Schmittgen TD (2001). Analysis of relative gene expression data using real-time quantitative PCR and the 2(-Delta Delta C(T)) Method. Methods.

[17] Zordoky BN, Anwar-Mohamed A, Aboutabl ME, El-Kadi AO (2010). Acute doxorubicin cardiotoxicity alters cardiac cytochrome P450 expression and arachidonic acid metabolism in rats. Toxicology and applied pharmacology.

[18] Althurwi HN, Maayah ZH, Elshenawy OH, El-Kadi AO (2015). Early Changes in Cytochrome P450s and Their Associated Arachidonic Acid Metabolites Play a Crucial Role in the Initiation of Cardiac Hypertrophy Induced by Isoproterenol. Drug metabolism and disposition: the biological fate of chemicals.

[19] El-Sherbeni AA, El-Kadi AO (2014). Characterization of arachidonic acid metabolism by rat cytochrome P450 enzymes: the involvement of CYP1As. Drug metabolism and disposition: the biological fate of chemicals.

[20] Liu Y, Peterson DA, Kimura H, Schubert D (1997). Mechanism of cellular 3-(4,5-dimethylthiazol-2-yl)-2,5-diphenyltetrazolium bromide (MTT) reduction. Journal of neurochemistry.

[21] Sambrook, J., Fritsch, E. F. & Maniatatis, T. In: Ford, N. (Ed.), Molecular Cloning. A Laboratory Manual. Cold Spring Harbour Laboratory Press, Plainview, NY (1989).

[22] Khan SR (2015). Proteomic profile of aminoglutethimide-induced apoptosis in HL-60 cells: Role of myeloperoxidase and arylamine free radicals. Chemico-biological interactions.

[23] Alsaikhan B, Fahlman R, Ding J, Tredget E, Metcalfe PD (2015). Proteomic profile of an acute partial bladder outlet obstruction. Canadian Urological Association journal = Journal de l’Association des urologues du Canada.

[24] Davidson, M. M. (Google Patents, 2007).

[25] Maayah, Z. H., Elshenawy, O. H., Althurwi, H. N., Abdelhamid, G. & El-Kadi, A. O. Human fetal ventricular cardiomyocyte, RL-14 cell line, is a promising model to study drug metabolizing enzymes and their associated arachidonic acid metabolites. *Journal of pharmacological and toxicological methods*, 10.1016/j.vascn.2014.11.005 (2014). 10.1016/j.vascn.2014.11.005 25454080

[26] Maayah ZH, Abdelhamid G, El-Kadi AO (2015). Development of cellular hypertrophy by 8-hydroxyeicosatetraenoic acid in the human ventricular cardiomyocyte, RL-14 cell line, is implicated by MAPK and NF-kappaB. Cell biology and toxicology.

[27] Maayah ZH, El Gendy MA, El-Kadi AO, Korashy HM (2013). Sunitinib, a tyrosine kinase inhibitor, induces cytochrome P450 1A1 gene in human breast cancer MCF7 cells through ligand-independent aryl hydrocarbon receptor activation. Archives of toxicology.

[28] Tse MM (2013). Cytochrome P450 epoxygenase metabolite, 14,15-EET, protects against isoproterenol-induced cellular hypertrophy in H9c2 rat cell line. Vascular pharmacology.

[29] Andrews NC, Faller DV (1991). A rapid micropreparation technique for extraction of DNA-binding proteins from limiting numbers of mammalian cells. Nucleic acids research.

[30] Bhattacharya N (2010). High-throughput detection of nuclear factor-kappaB activity using a sensitive oligo-based chemiluminescent enzyme-linked immunosorbent assay. International journal of cancer. Journal international du cancer.

[31] Barchiesi F (2002). Methoxyestradiols mediate estradiol-induced antimitogenesis in human aortic SMCs. Hypertension.

[32] Tevaarwerk AJ (2009). Phase I trial of 2-methoxyestradiol NanoCrystal dispersion in advanced solid malignancies. Clinical cancer research: an official journal of the American Association for Cancer Research.

[33] Ni L (2011). beta-AR blockers suppresses ER stress in cardiac hypertrophy and heart failure. PLoS One.

[34] Qi G (2011). Angiotensin II infusion-induced inflammation, monocytic fibroblast precursor infiltration, and cardiac fibrosis are pressure dependent. Cardiovascular toxicology.

[35] Matsui Y (2004). Role of osteopontin in cardiac fibrosis and remodeling in angiotensin II-induced cardiac hypertrophy. Hypertension.

[36] Siddiq T, Richardson PJ, Trotter SE, Preedy VR (1996). Protein synthesis during regression of left ventricular hypertrophy with lisinopril in abdominal aortic constriction model of hypertension. Biochemical and molecular medicine.

[37] Brahn E (2008). An angiogenesis inhibitor, 2-methoxyestradiol, involutes rat collagen-induced arthritis and suppresses gene expression of synovial vascular endothelial growth factor and basic fibroblast growth factor. The Journal of rheumatology.

[38] Plum SM (2009). Disease modifying and antiangiogenic activity of 2-methoxyestradiol in a murine model of rheumatoid arthritis. BMC musculoskeletal disorders.

[39] Bonacasa B (2008). 2-Methoxyestradiol attenuates hypertension and coronary vascular remodeling in spontaneously hypertensive rats. Maturitas.

[40] Yuan W (2013). Estrogen metabolite 2-methoxyestradiol prevents hypertension in deoxycorticosterone acetate-salt rats. Cardiovascular drugs and therapy/sponsored by the International Society of Cardiovascular Pharmacotherapy.

[41] Dubey RK, Gillespie DG, Jackson EK, Keller PJ (1998). 17Beta-estradiol, its metabolites, and progesterone inhibit cardiac fibroblast growth. Hypertension.

[42] Asara JM, Christofk HR, Freimark LM, Cantley LC (2008). A label-free quantification method by MS/MS TIC compared to SILAC and spectral counting in a proteomics screen. Proteomics.

[43] Smith SJ (2016). The cardiac-restricted protein ADP-ribosylhydrolase-like 1 is essential for heart chamber outgrowth and acts on muscle actin filament assembly. Developmental biology.

[44] Ho JE (2012). Galectin-3, a marker of cardiac fibrosis, predicts incident heart failure in the community. Journal of the American College of Cardiology.

[45] Balasubramanian A (2014). Fam65b is important for formation of the HDAC6-dysferlin protein complex during myogenic cell differentiation. FASEB journal: official publication of the Federation of American Societies for Experimental Biology.

[46] Vasile VC, Edwards WD, Ommen SR, Ackerman MJ (2006). Obstructive hypertrophic cardiomyopathy is associated with reduced expression of vinculin in the intercalated disc. Biochemical and biophysical research communications.

[47] Dubey RK, Tofovic SP, Jackson EK (2004). Cardiovascular pharmacology of estradiol metabolites. The Journal of pharmacology and experimental therapeutics.

[48] Hayakawa Y (2003). Inhibition of cardiac myocyte apoptosis improves cardiac function and abolishes mortality in the peripartum cardiomyopathy of Galpha(q) transgenic mice. Circulation.

[49] van Empel VP (2005). Myocyte apoptosis in heart failure. Cardiovascular research.

[50] Brown JW, Kesler CT, Neary T, Fishman LM (2001). Effects of androgens and estrogens and catechol and methoxy-estrogen derivatives on mitogen-activated protein kinase (ERK(1,2)) activity in SW-13 human adrenal carcinoma cells. Hormone and metabolic research = Hormon- und Stoffwechselforschung = Hormones et metabolisme.

[51] Prossnitz ER, Arterburn JB, Sklar LA (2007). GPR30: A G protein-coupled receptor for estrogen. Molecular and cellular endocrinology.

[52] Lopaschuk GD, Ussher JR, Folmes CD, Jaswal JS, Stanley WC (2010). Myocardial fatty acid metabolism in health and disease. Physiological reviews.

[53] Desvergne B, Michalik L, Wahli W (2006). Transcriptional regulation of metabolism. Physiological reviews.

[54] Piao L (2010). The inhibition of pyruvate dehydrogenase kinase improves impaired cardiac function and electrical remodeling in two models of right ventricular hypertrophy: resuscitating the hibernating right ventricle. Journal of molecular medicine.

[55] Atherton HJ (2011). Role of pyruvate dehydrogenase inhibition in the development of hypertrophy in the hyperthyroid rat heart: a combined magnetic resonance imaging and hyperpolarized magnetic resonance spectroscopy study. Circulation.

[56] Barchiesi F (2006). 2-Methoxyestradiol, an estradiol metabolite, inhibits neointima formation and smooth muscle cell growth via double blockade of the cell cycle. Circulation research.

[57] Dingli D, Timm M, Russell SJ, Witzig TE, Rajkumar SV (2002). Promising preclinical activity of 2-methoxyestradiol in multiple myeloma. Clinical cancer research: an official journal of the American Association for Cancer Research.

[58] Sibonga JD (2003). Dose-response effects of 2-methoxyestradiol on estrogen target tissues in the ovariectomized rat. Endocrinology.

[59] Yorifuji T (2011). 2-Methoxyestradiol ameliorates glucose tolerance with the increase in beta-cell mass in db/db mice. Journal of diabetes investigation.

[60] Tofovic SP, Dubey RK, Jackson EK (2001). 2-Hydroxyestradiol attenuates the development of obesity, the metabolic syndrome, and vascular and renal dysfunction in obese ZSF1 rats. The Journal of pharmacology and experimental therapeutics.

[61] Maulik SK, Kumar S (2012). Oxidative stress and cardiac hypertrophy: a review. Toxicology mechanisms and methods.

[62] Seeger H, Mueck AO, Lippert TH (1997). Effect of estradiol metabolites on the susceptibility of low density lipoprotein to oxidation. Life sciences.

[63] Molojavyi A (2010). Myoglobin-deficient mice activate a distinct cardiac gene expression program in response to isoproterenol-induced hypertrophy. Physiological genomics.

[64] Osadchii OE (2007). Cardiac hypertrophy induced by sustained beta-adrenoreceptor activation: pathophysiological aspects. Heart failure reviews.

[65] Meszaros J, Levai G (1990). Ultrastructural and electrophysiological alterations during the development of catecholamine-induced cardiac hypertrophy and failure. Acta biologica Hungarica.

