# Supplementary material for: 2-Methoxyestradiol protects against pressure overload-induced left ventricular hypertrophy
Source: Sci Rep. 2018 Feb 9;8:2780. doi: 10.1038/s41598-018-20613-9 (PMC5807528; doi:10.1038/s41598-018-20613-9)
Supplement: Supplementary file 1 — Supplementary Figures [file 41598_2018_20613_MOESM1_ESM.pdf]

2-Methoxyestradiol protects against pressure overload-induced left ventricular hypertrophy

Zaid H. Maayah[1], Jody Levasseur[2], Ramanaguru Siva Piragasam[3], Ghada Abdelhamid[1], Jason R. B. Dyck[2], Richard P. Fahlman[3,4], Arno G. Siraki[1] and Ayman O.S. El-Kadi[1*]

Supplementary Figures: 1-21 Uncropped full-length blots

Supplementary Figure 15: Effect of 2ME on mid-chain HETE level and the expression of CYP1B1, LOXs and COX protein

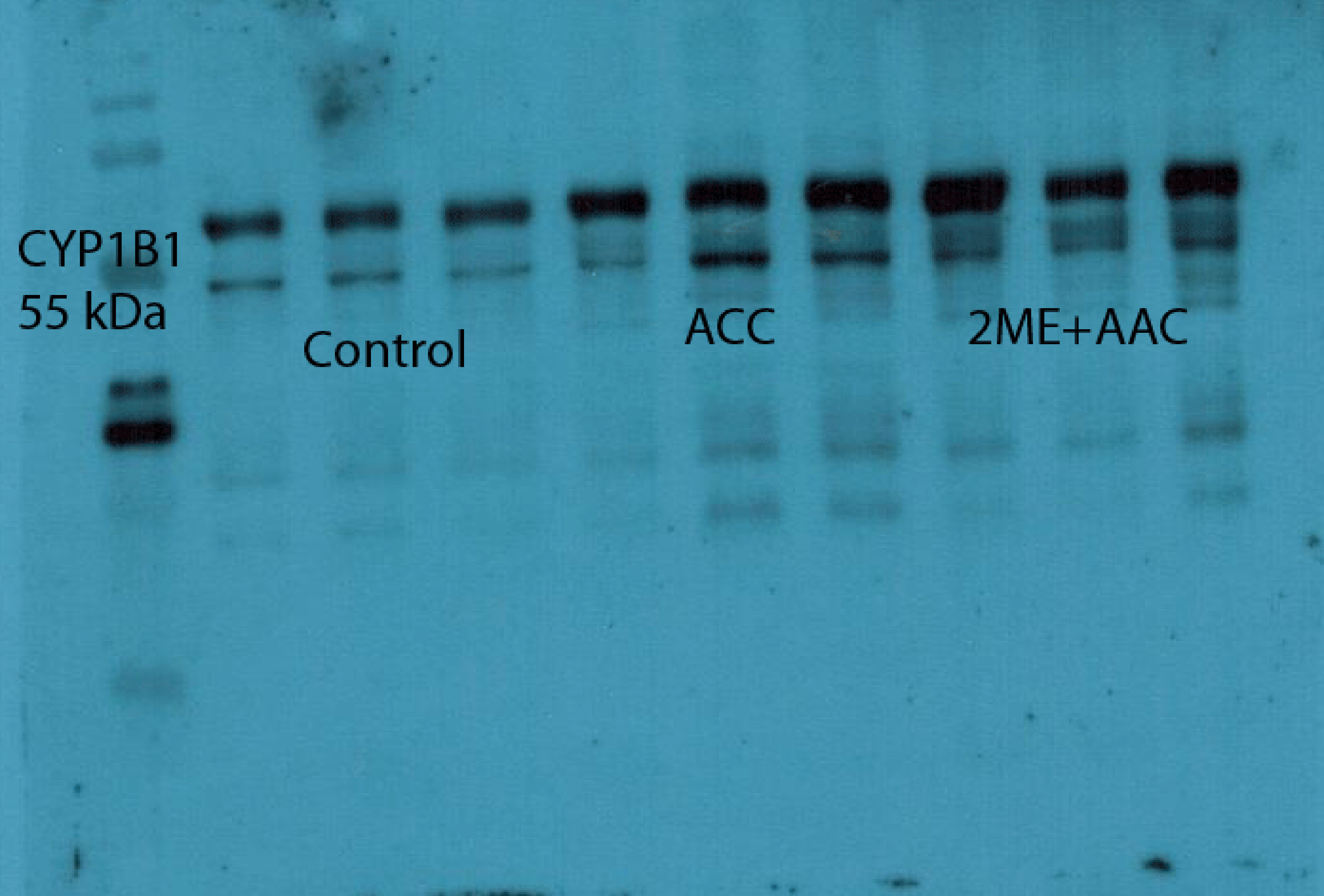
CYP1B1
55 kDa
Control
ACC
2ME+AAC

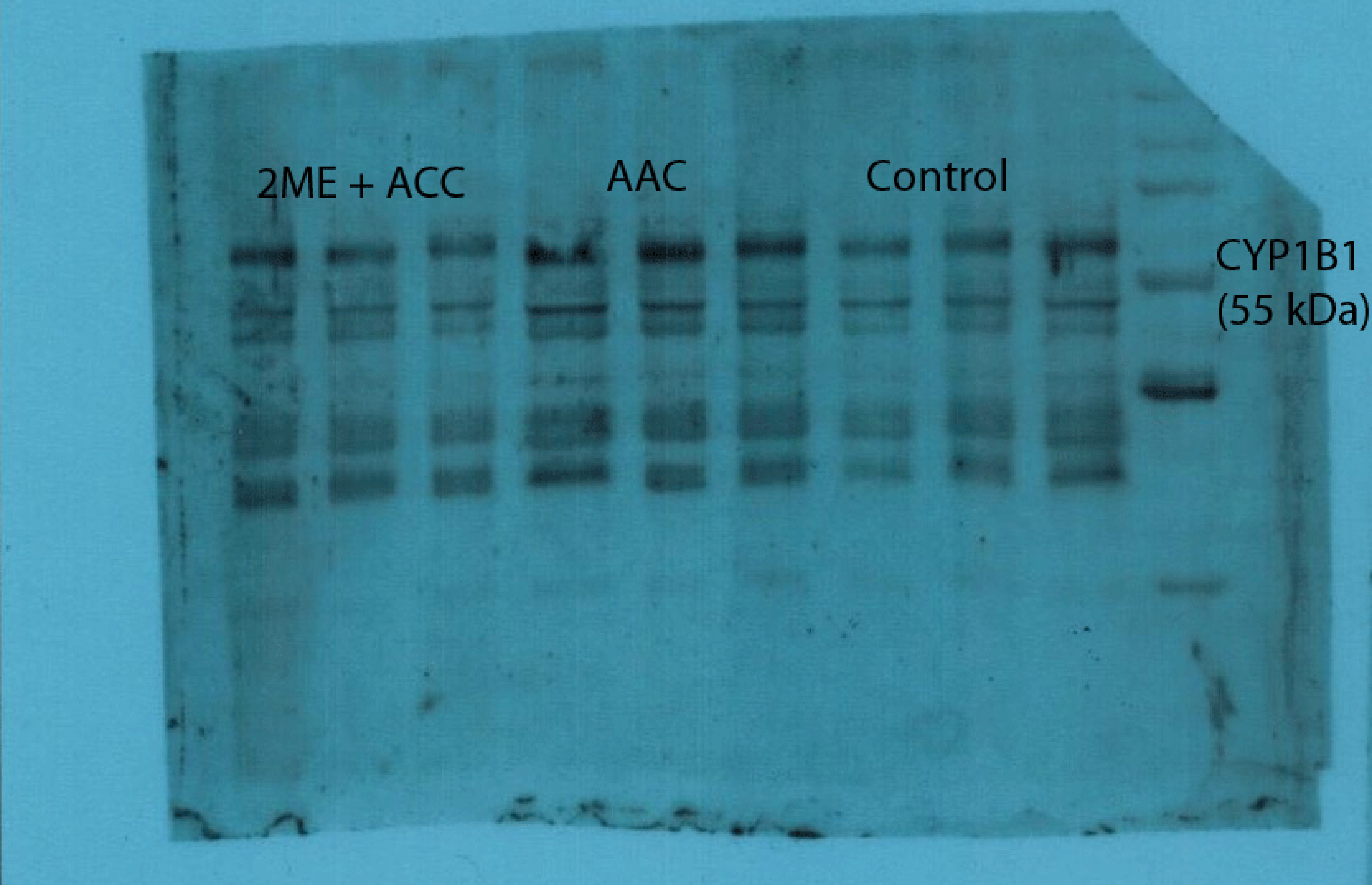
2ME + ACC
AAC
Control
CYP1B1
(55 kDa)

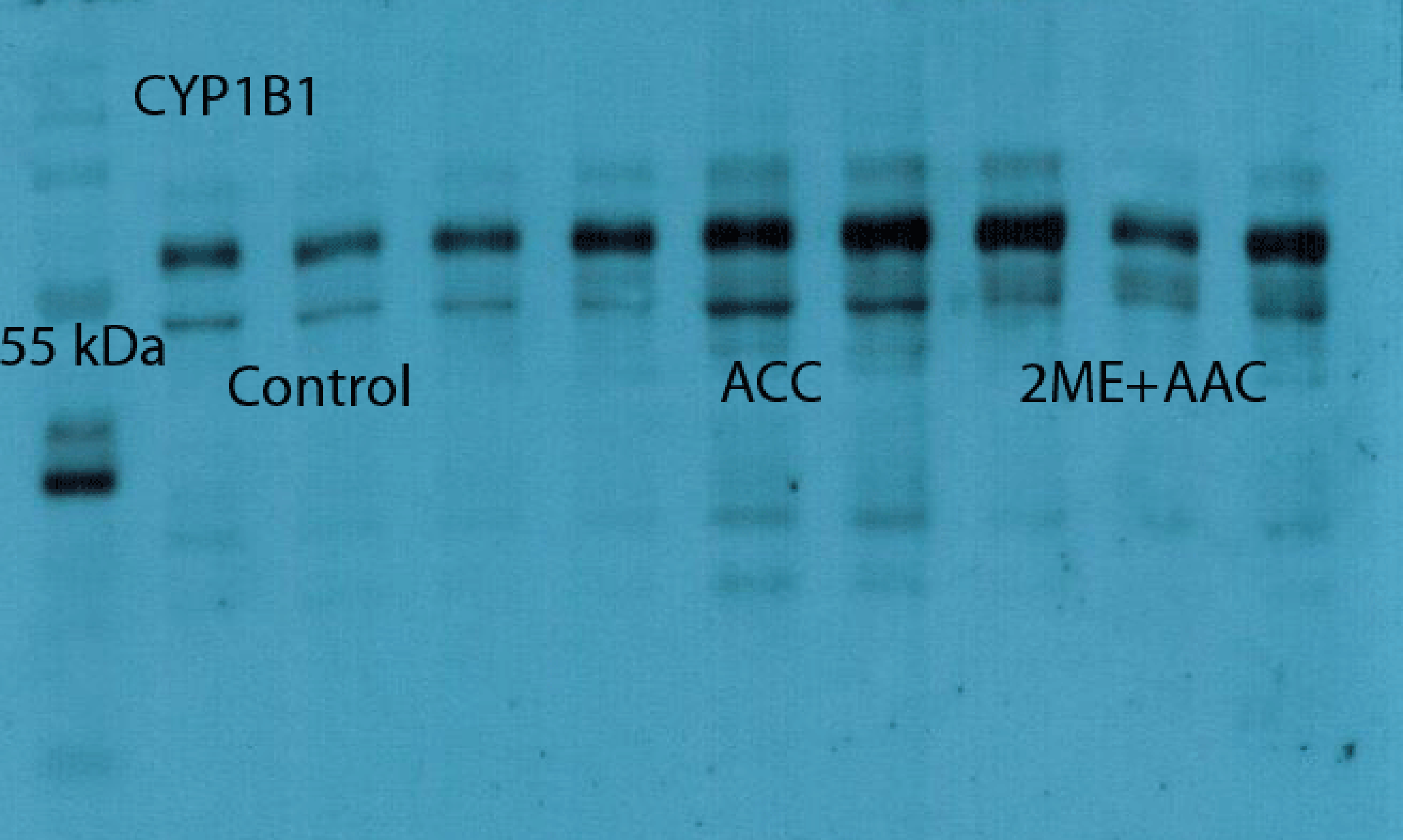
CYP1B1
55 kDa
Control
ACC
2ME+AAC

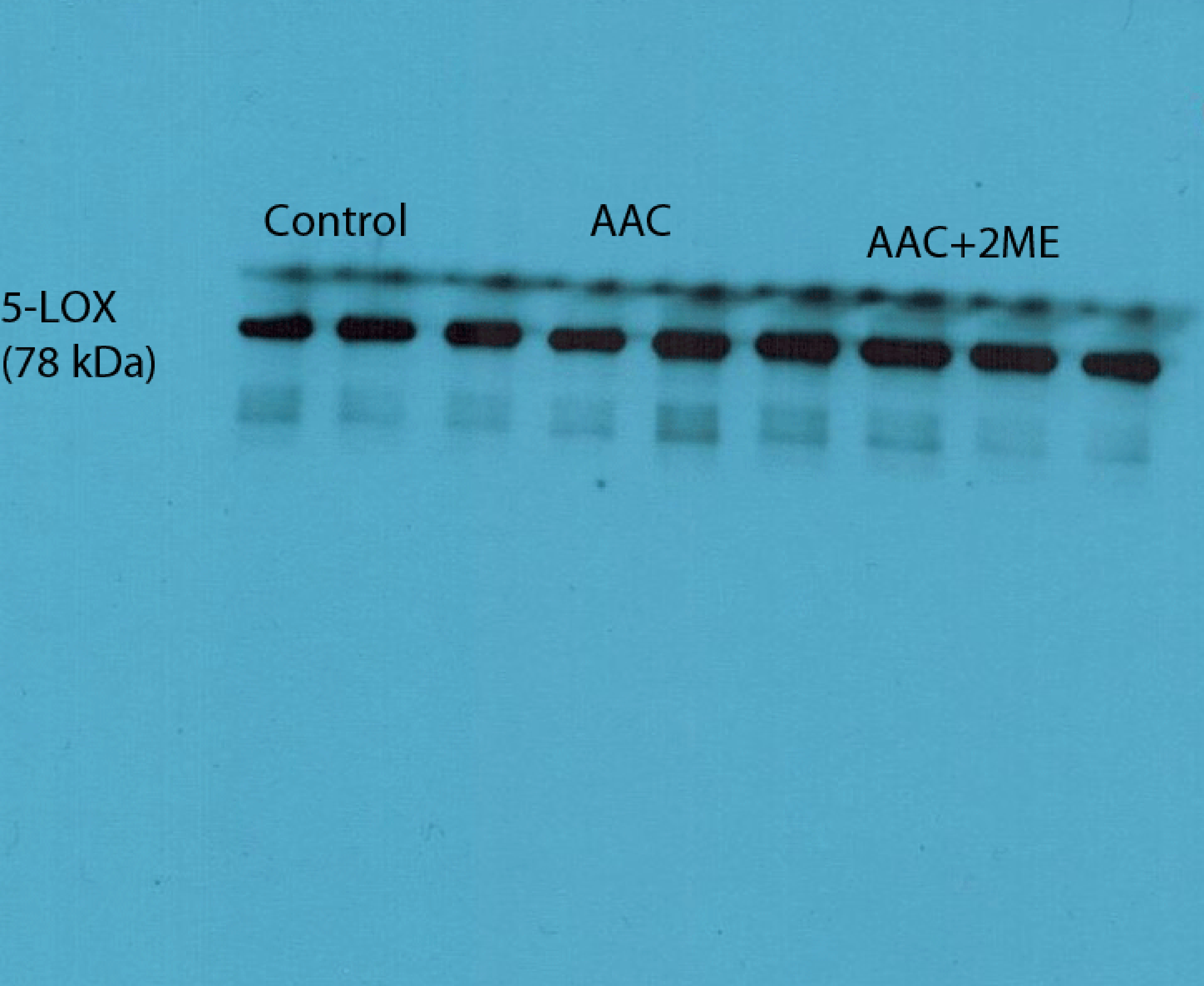

Control
AAC
AAC+2ME
5-LOX
(78 kDa)

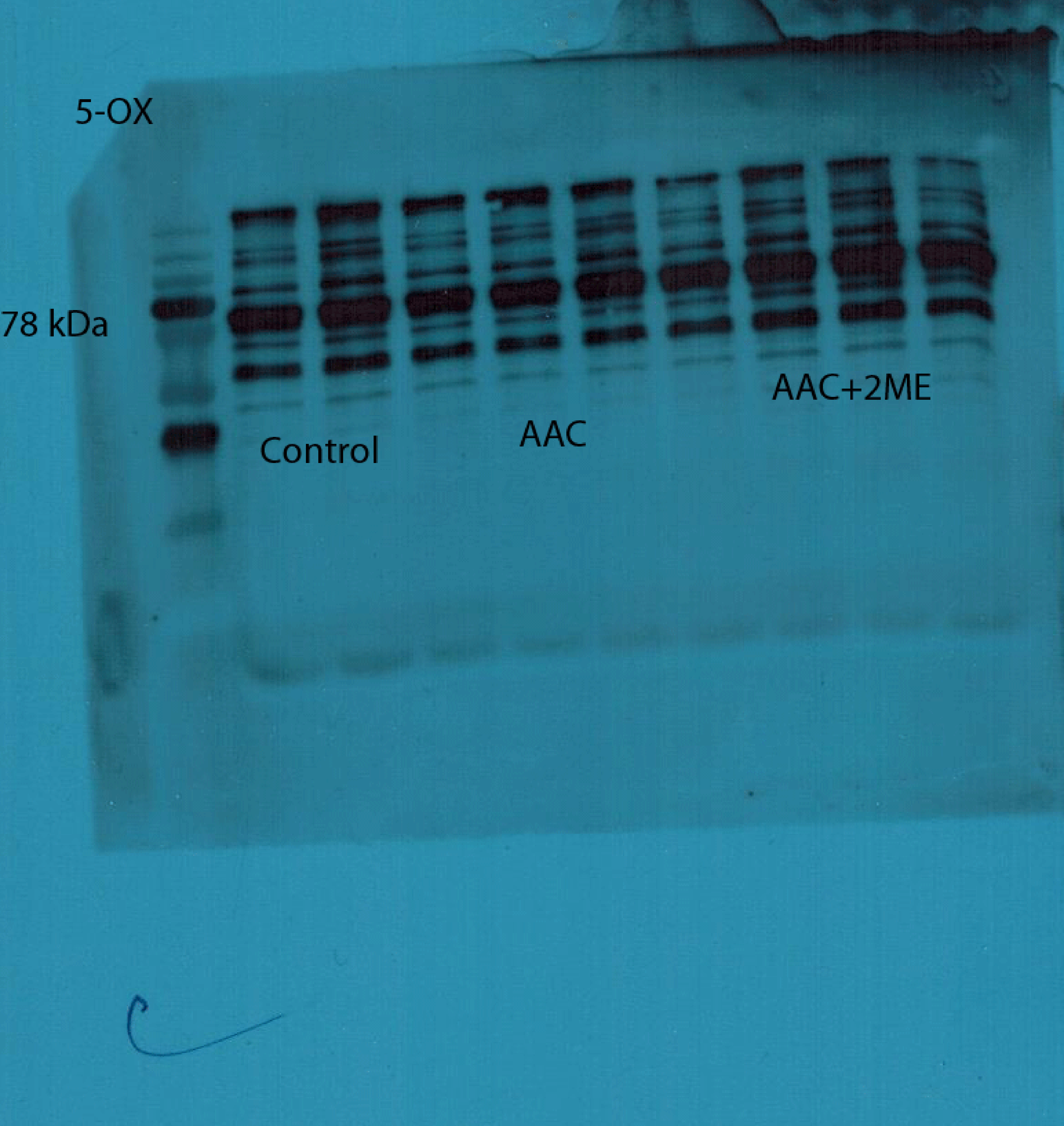
5-OX
78 kDa
Control
AAC
AAC+2ME

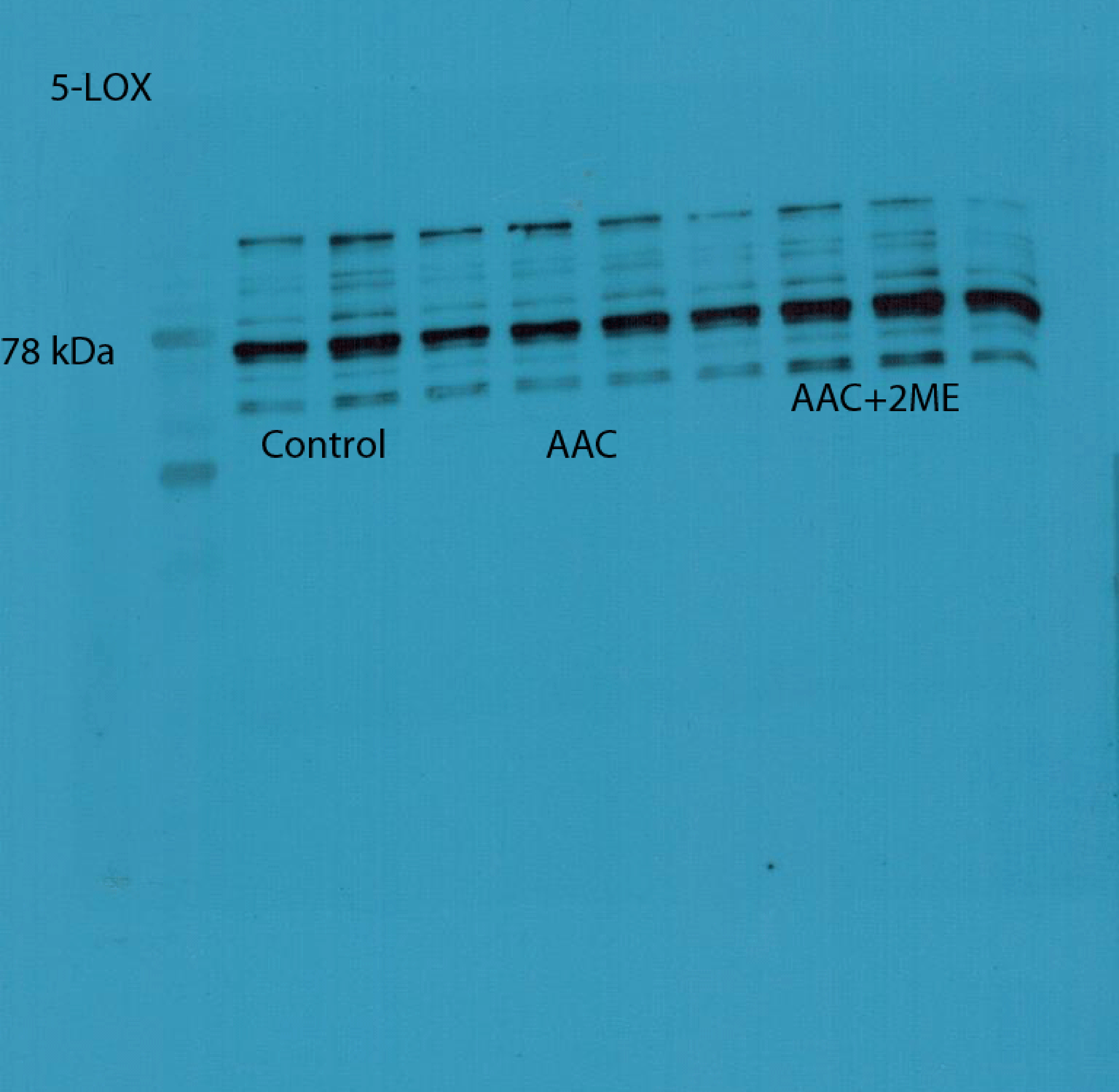
5-LOX
78 kDa
Control
AAC
AAC+2ME

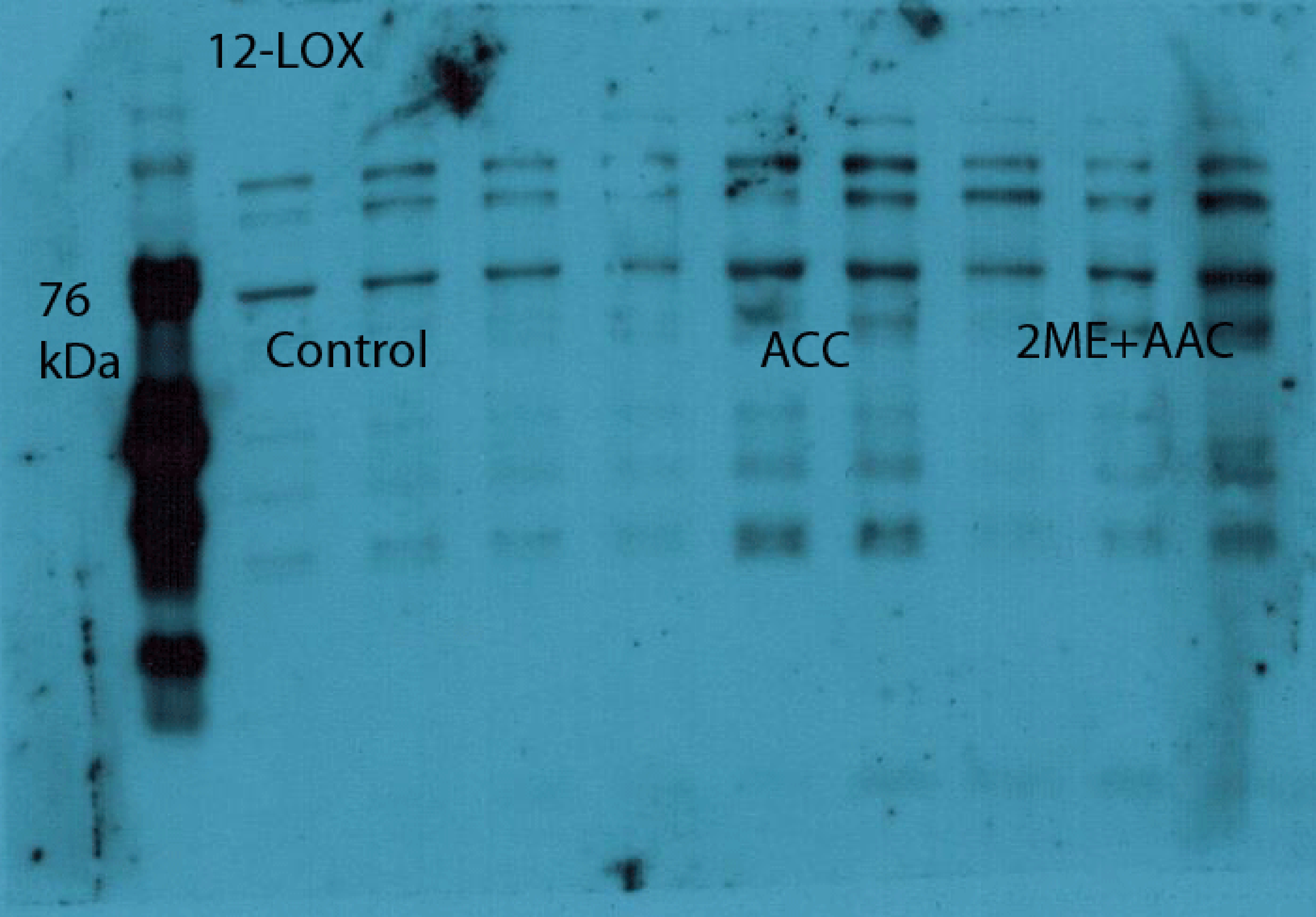
12-LOX
76
kDa
Control
ACC
2ME+AAC

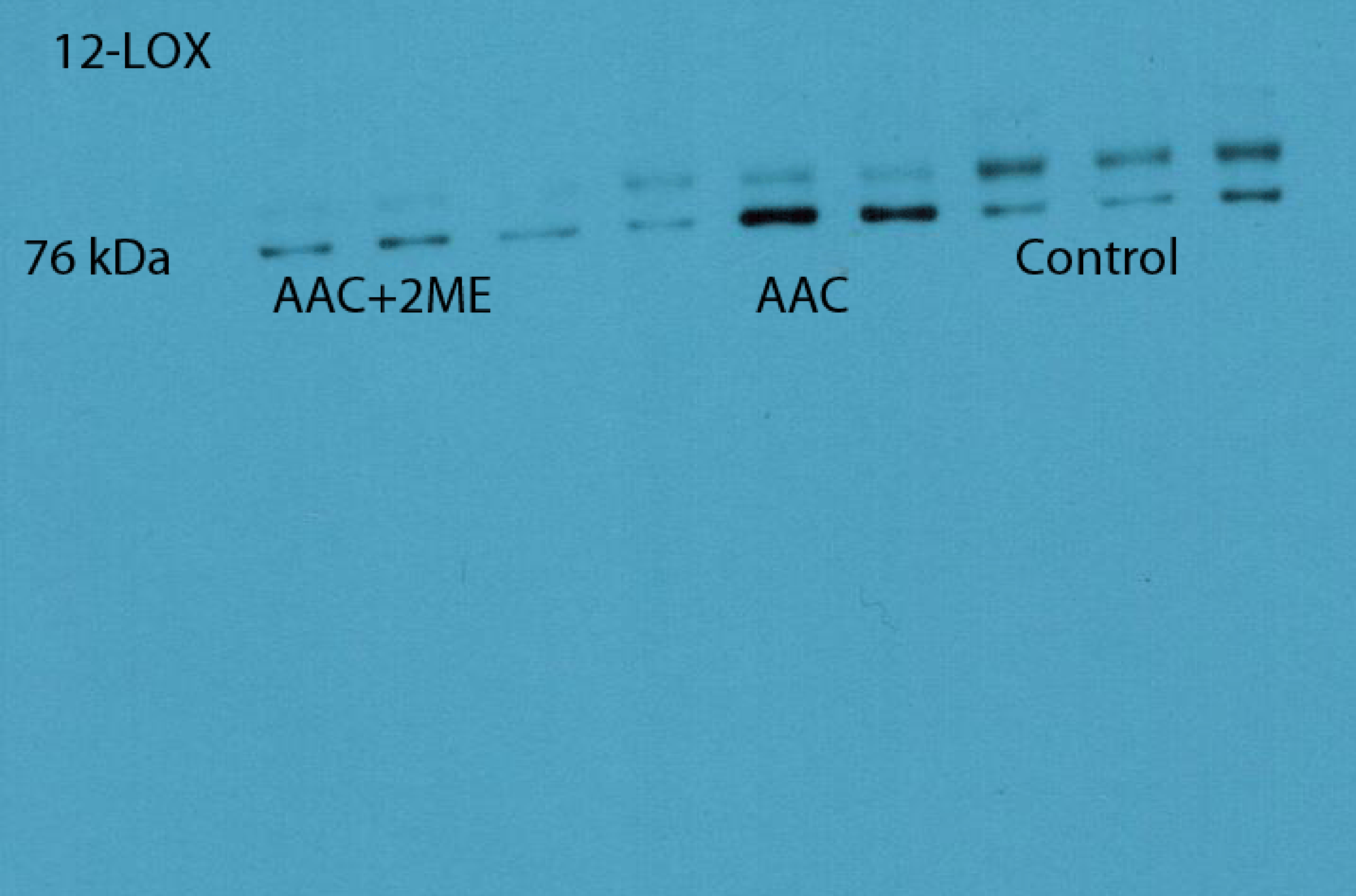
12-LOX
76 kDa
AAC+2ME
AAC
Control

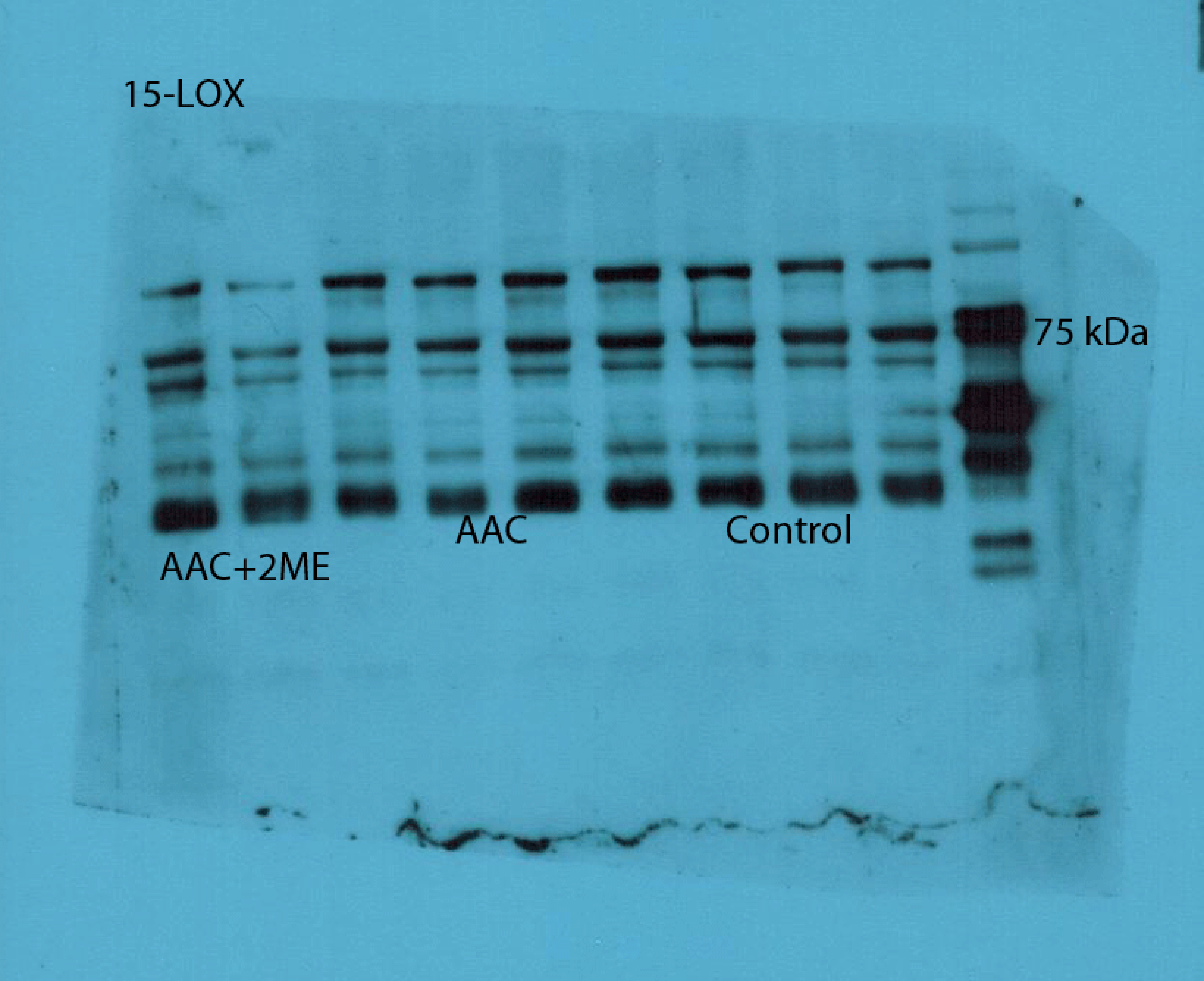
15-LOX
75 kDa
AAC+2ME
AAC
Control

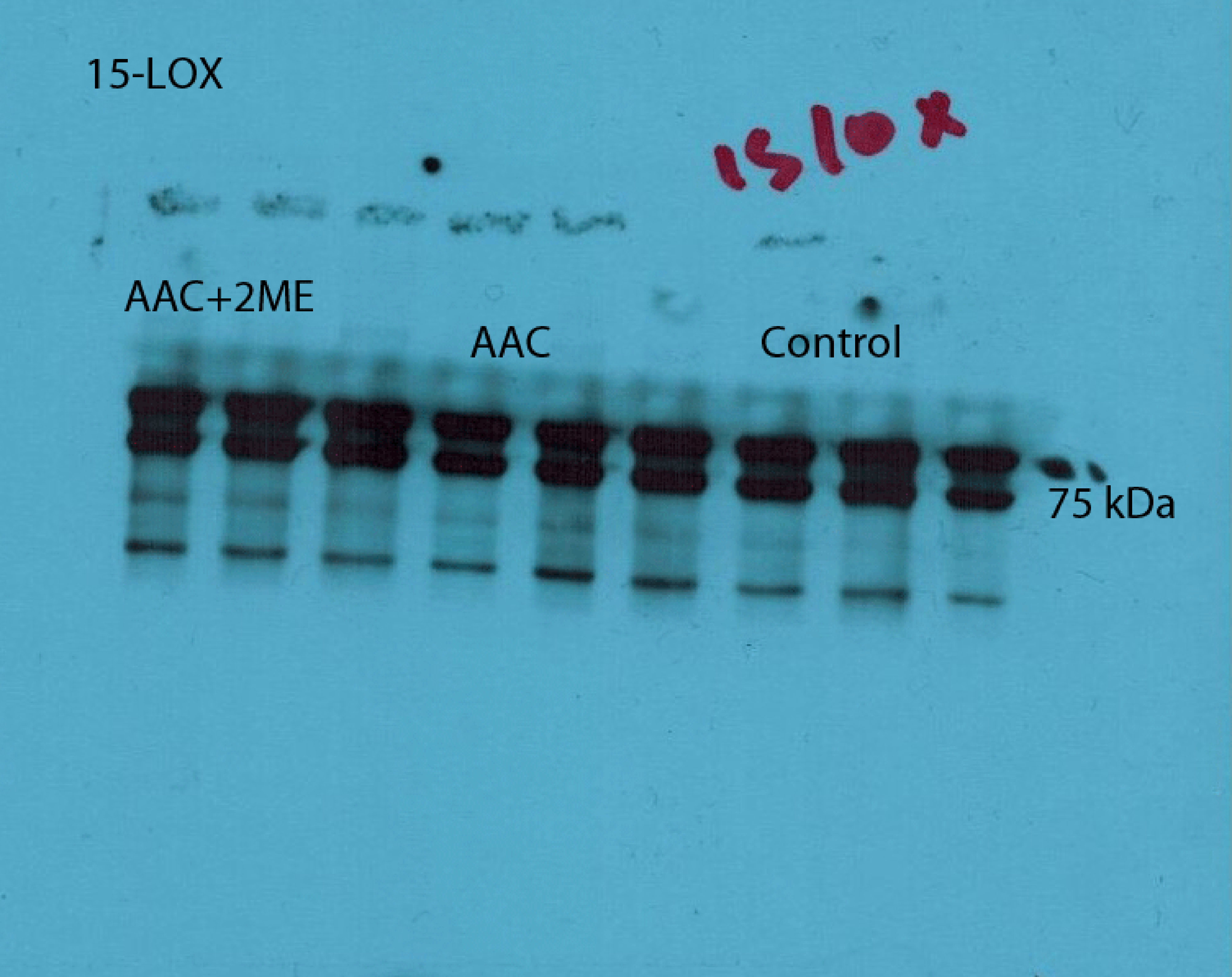
15-LOX
15lox
AAC+2ME
AAC
Control
75 kDa

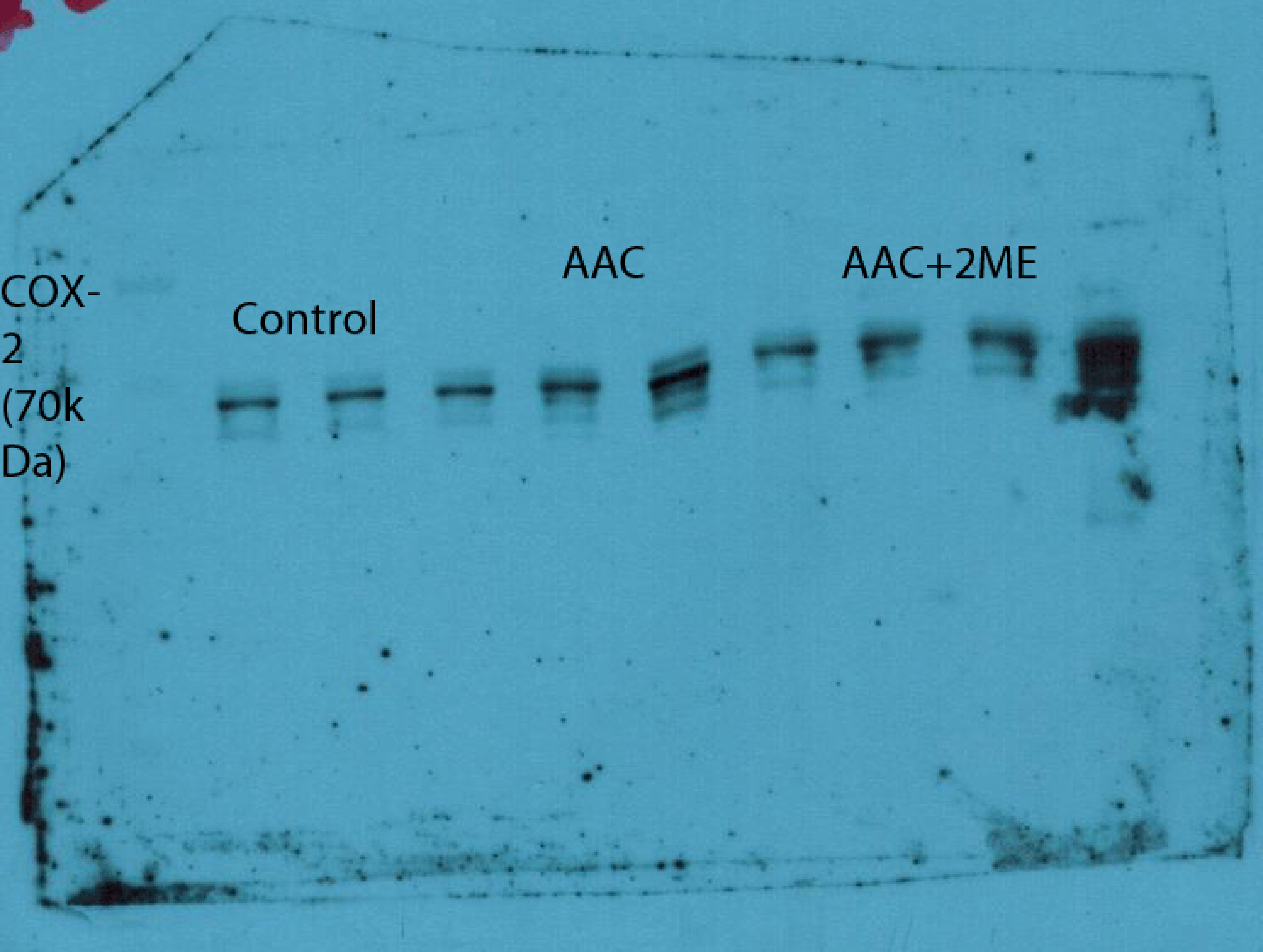
COX-2 (70k Da)
Control
AAC
AAC+2ME

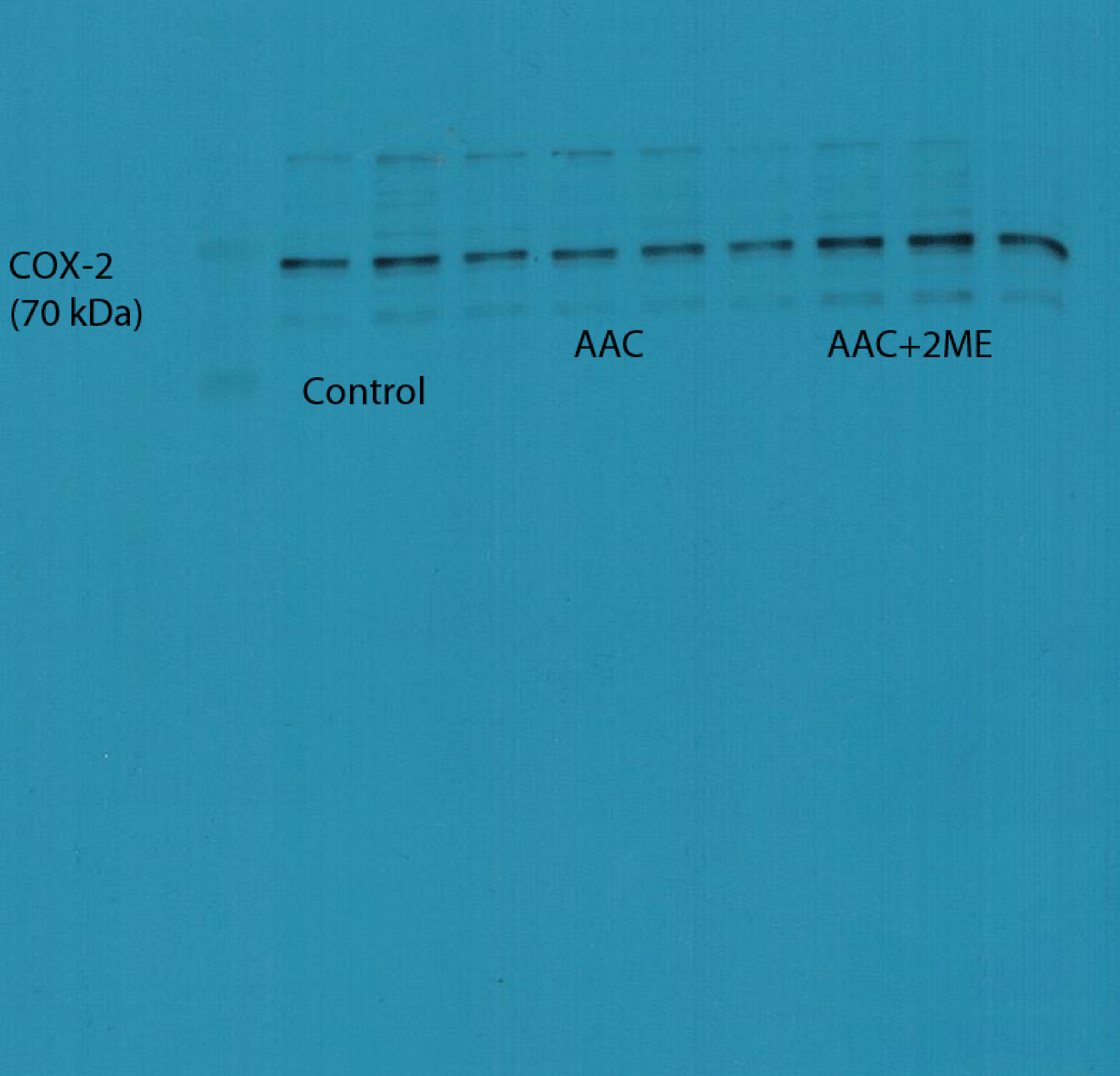
COX-2
(70 kDa)
AAC
AAC+2ME
Control

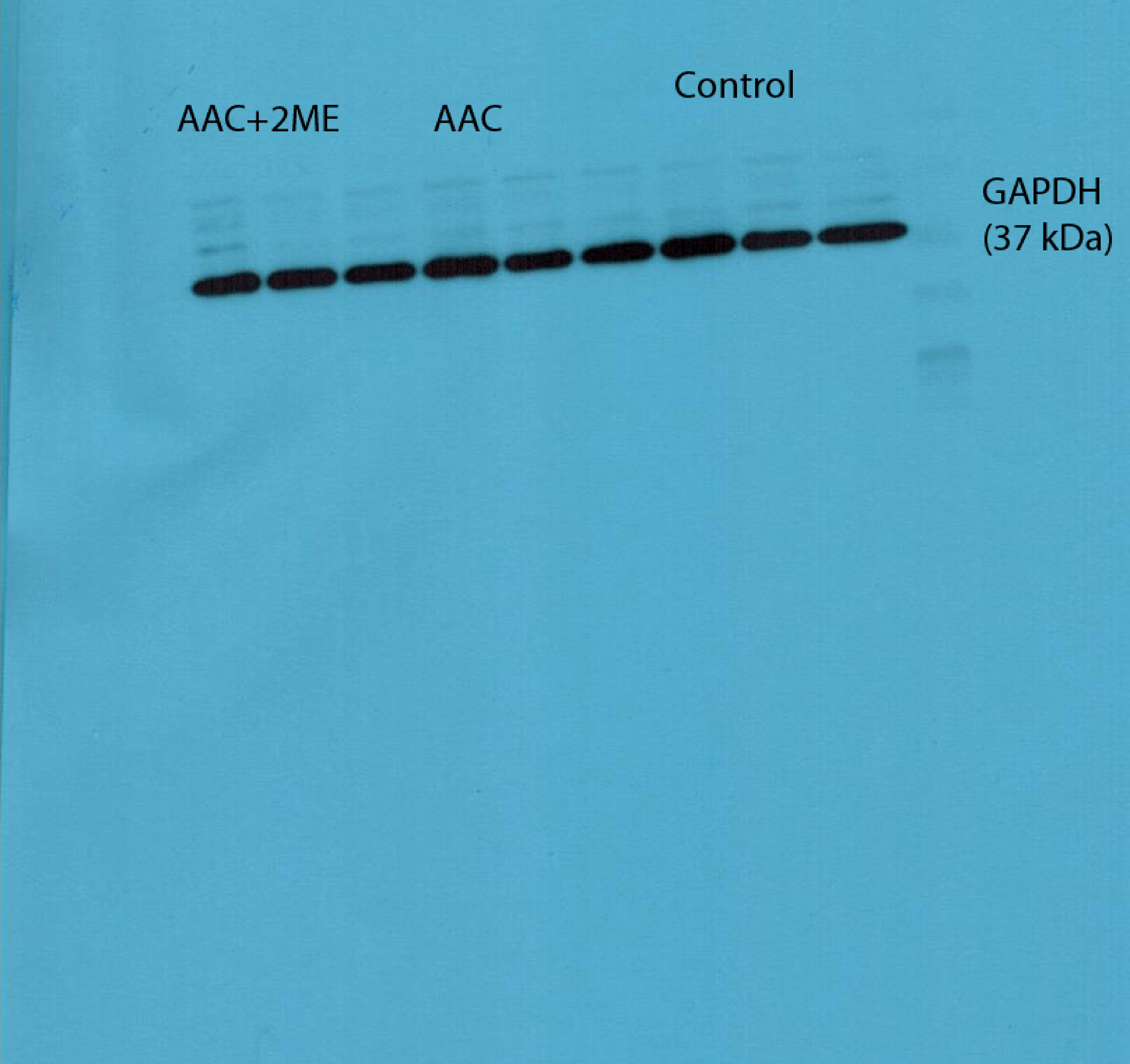
AAC+2ME
AAC
Control
GAPDH
(37 kDa)

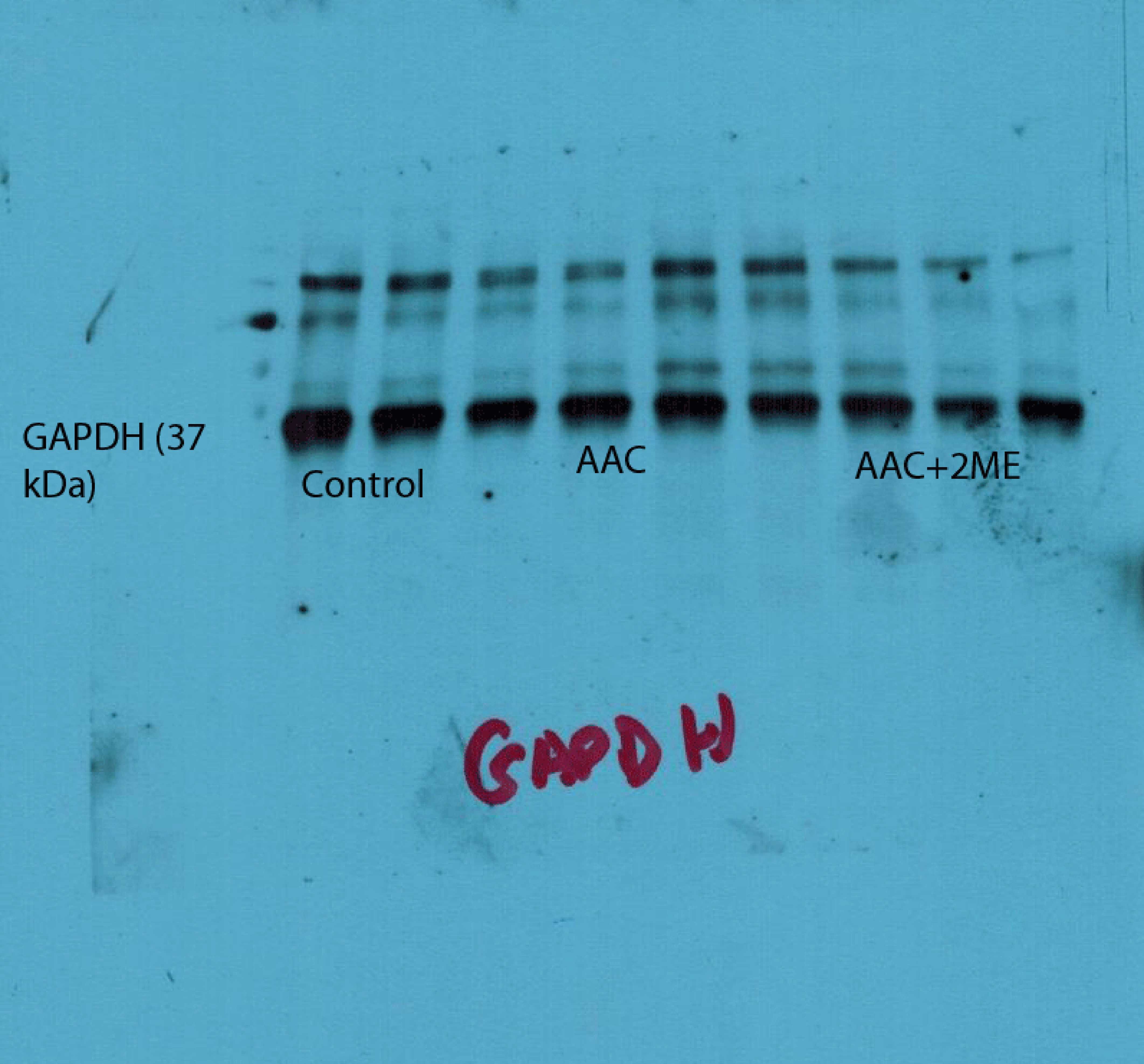
GAPDH (37 kDa)
Control
AAC
AAC+2ME
GAPDH

Supplementary Figure 15

A)

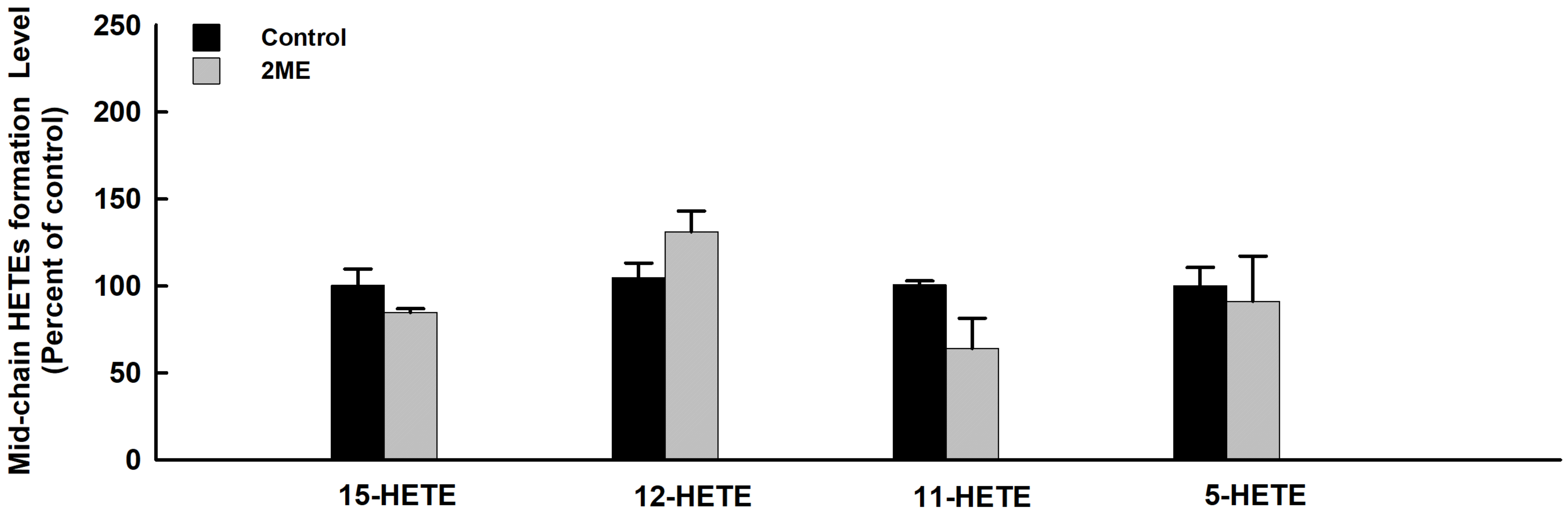

B)

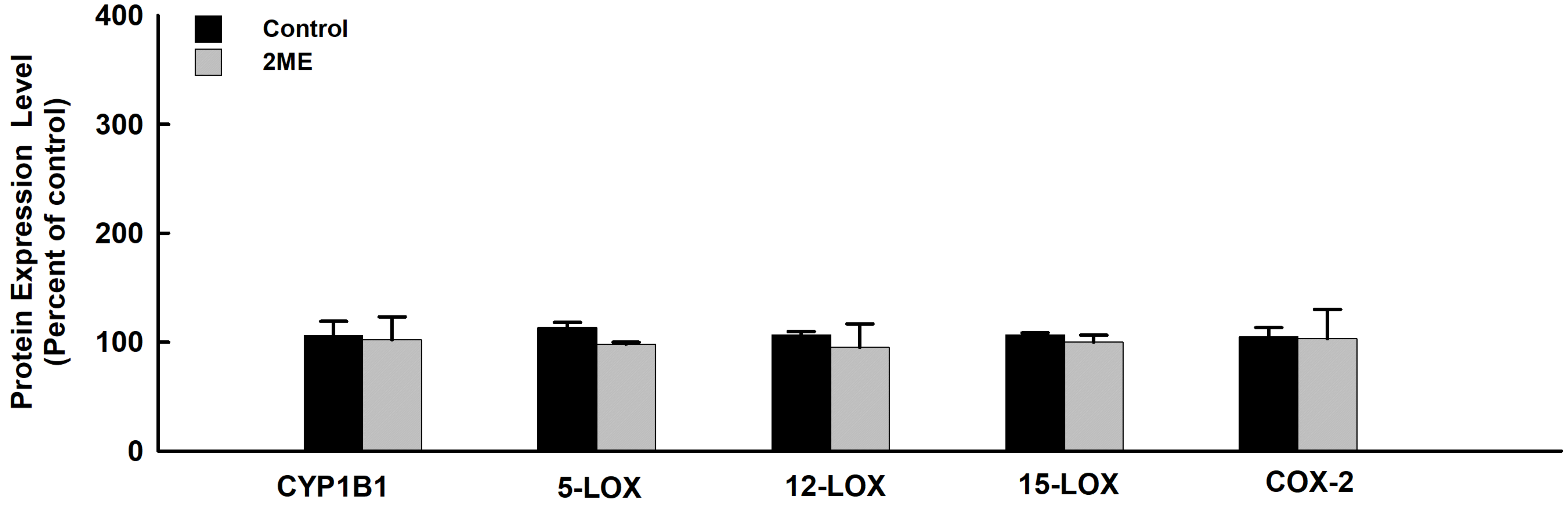

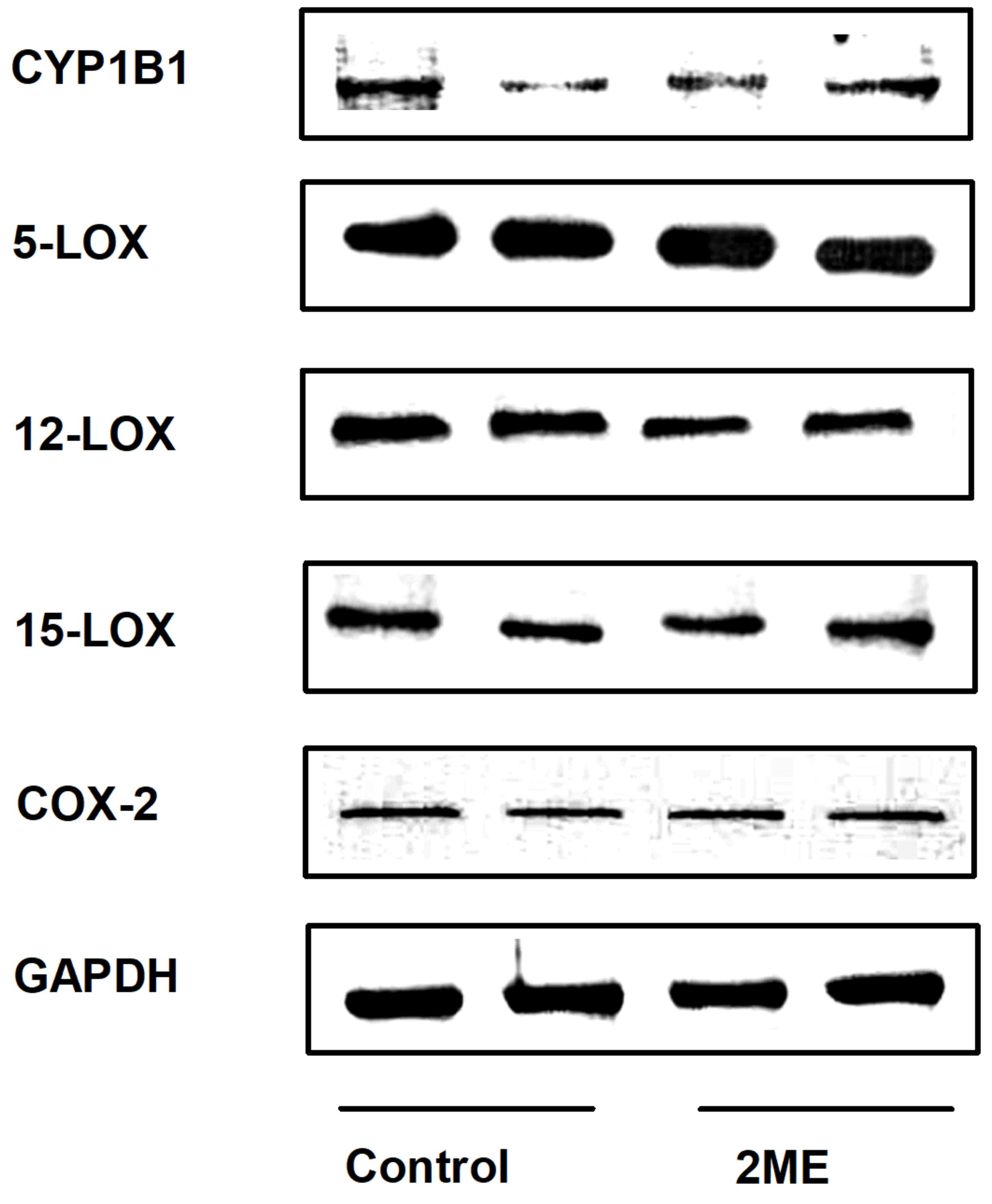

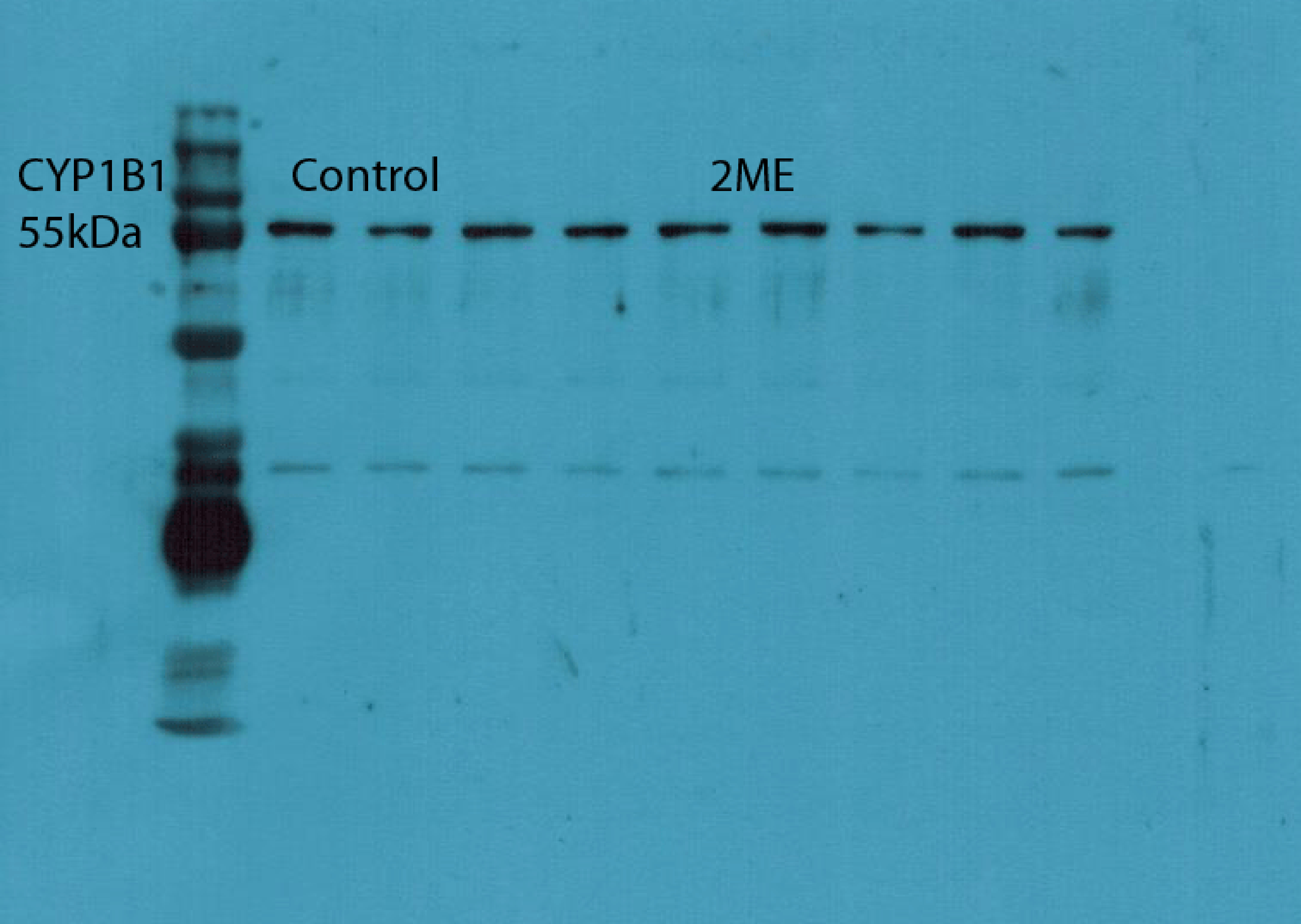
CYP1B1
55kDa
Control
2ME

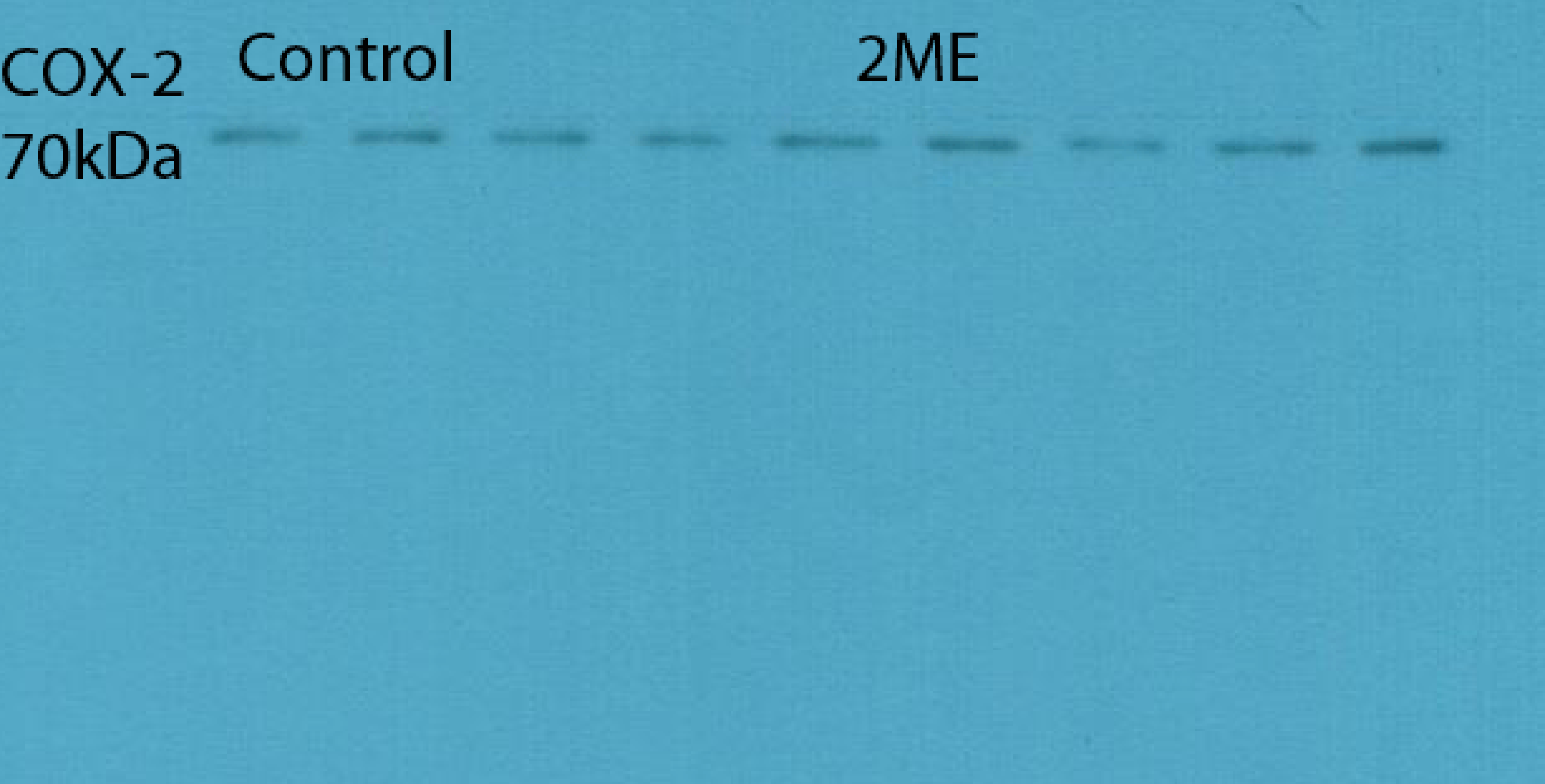
COX-2
70kDa
Control
2ME

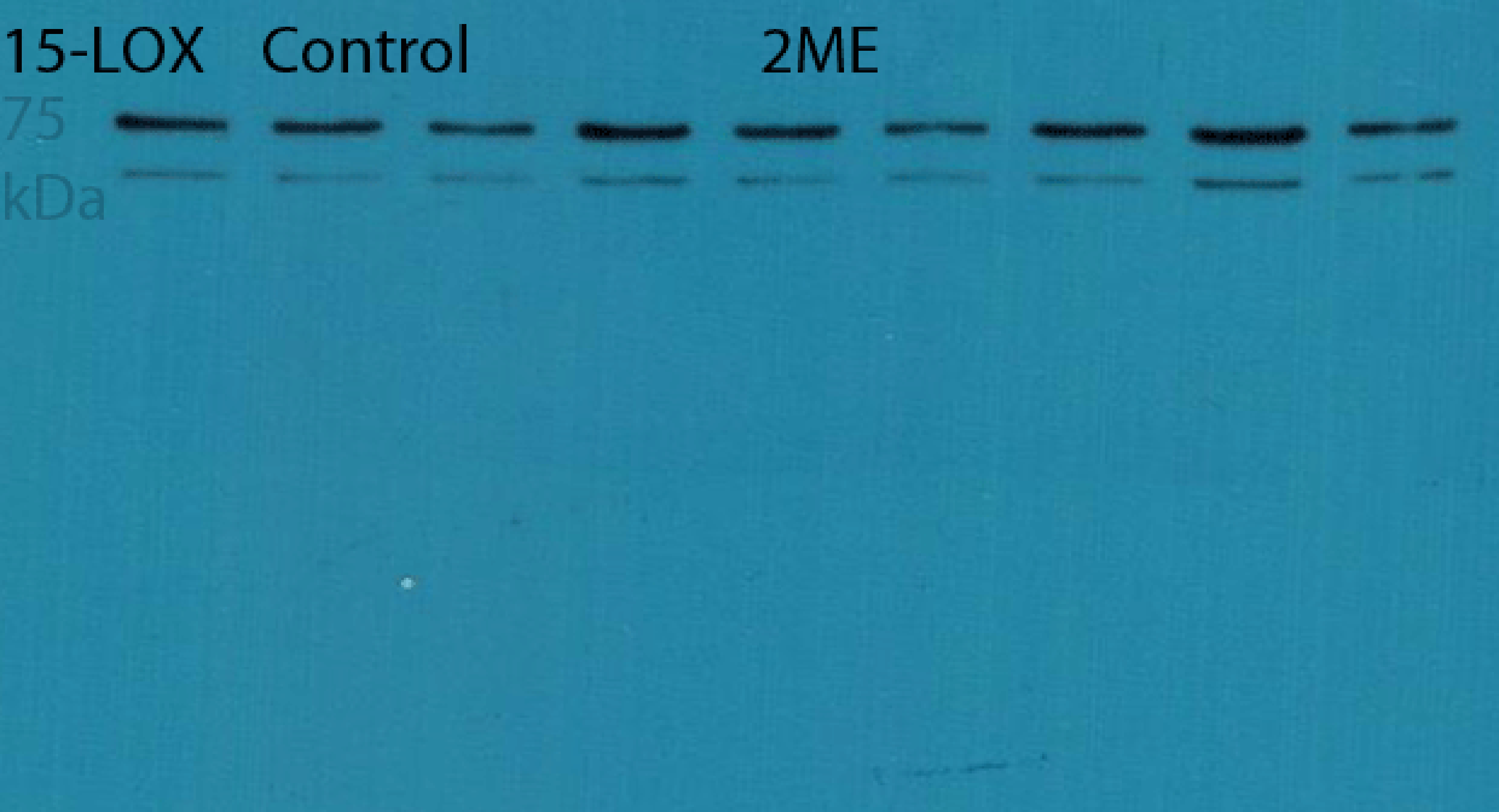
15-LOX
Control
2ME
75
kDa

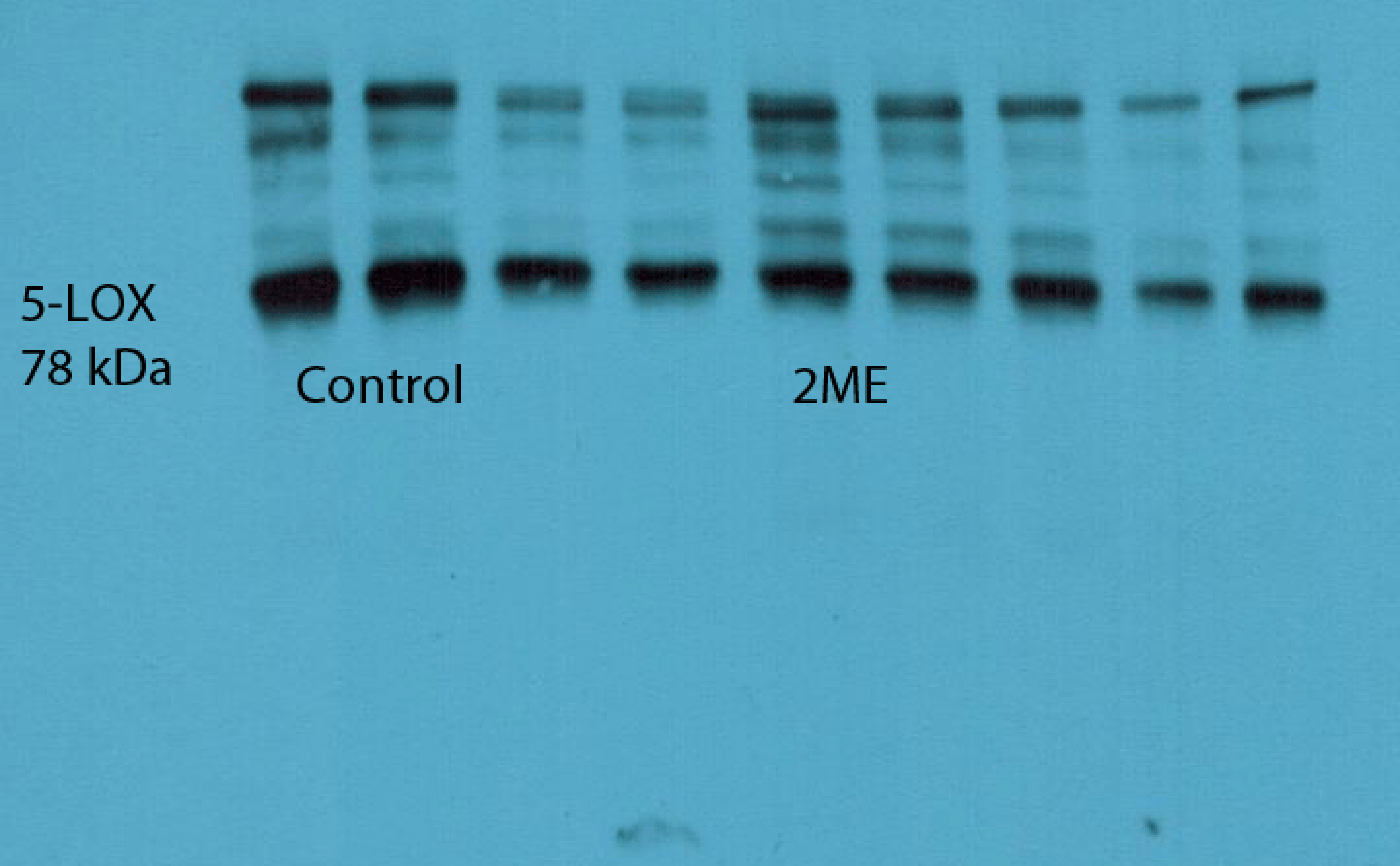
5-LOX
78 kDa
Control
2ME

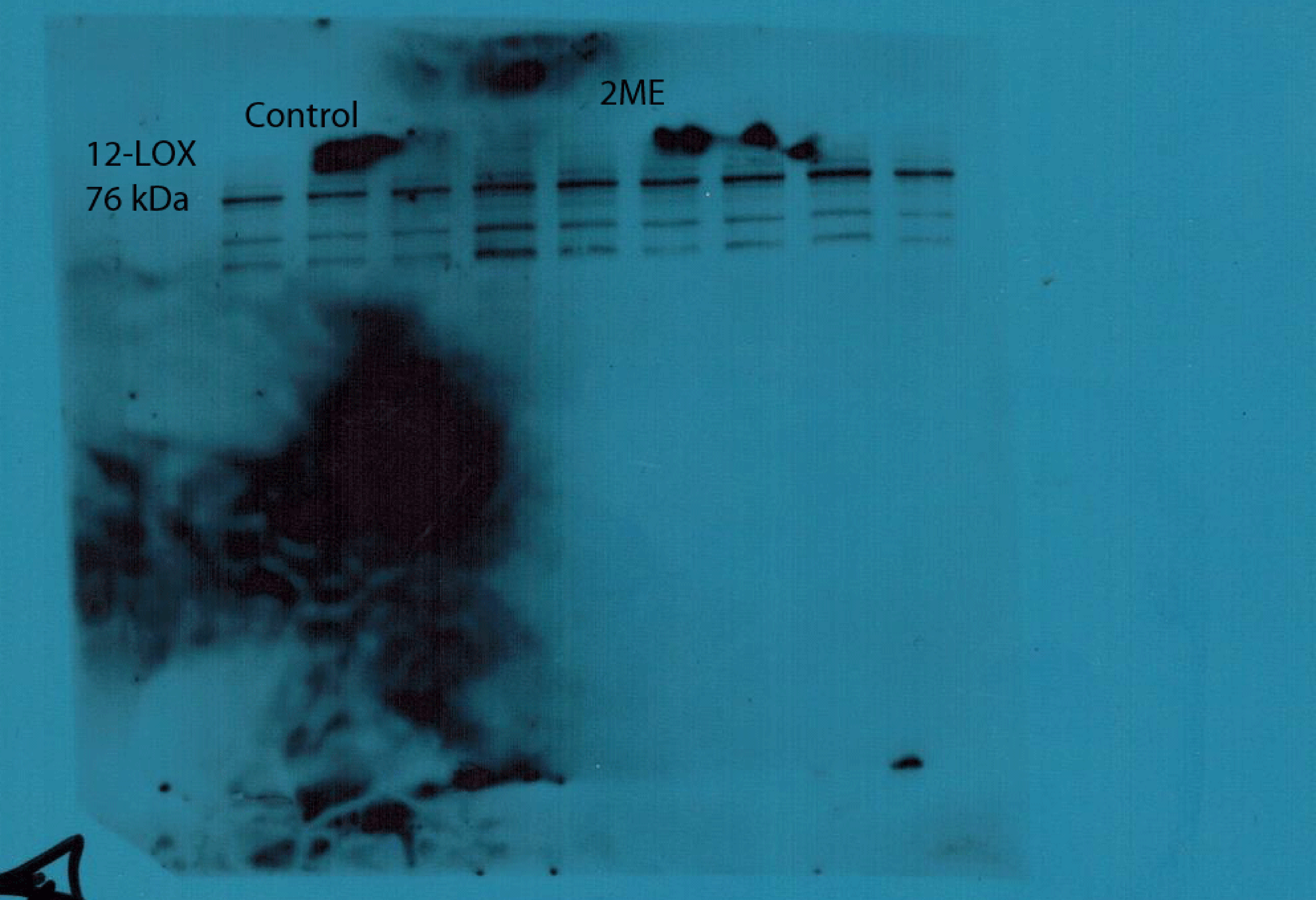

12-LOX
76 kDa
Control
2ME

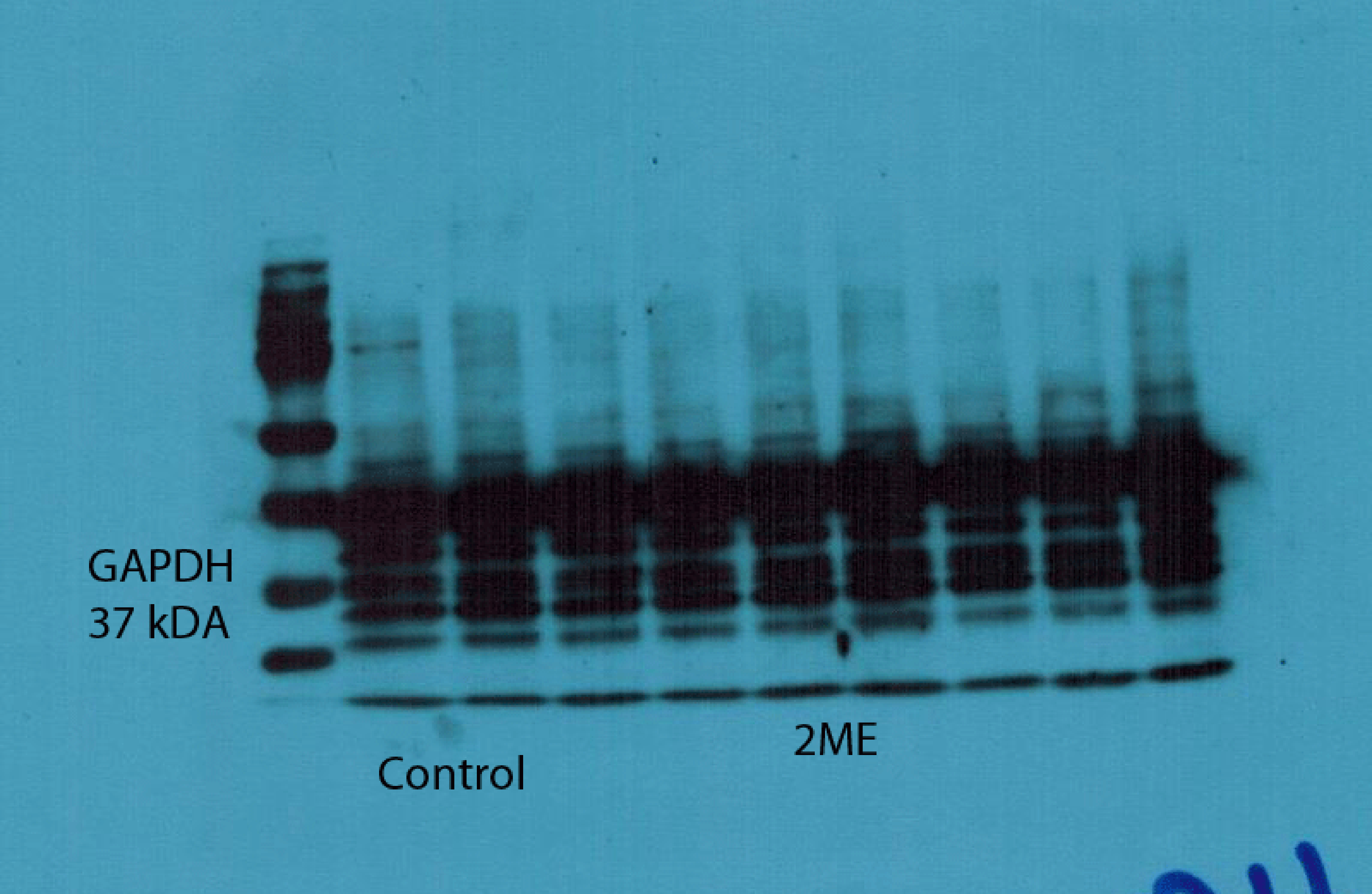
GAPDH
37 kDA
Control
2ME
